# Serine synthesis and catabolism in starved lung cancer and primary bronchial epithelial cells

**DOI:** 10.1186/s40170-024-00337-3

**Published:** 2024-03-21

**Authors:** Theresa Haitzmann, Katharina Schindlmaier, Tobias Frech, Ayusi Mondal, Visnja Bubalo, Barbara Konrad, Gabriele Bluemel, Philipp Stiegler, Stefanie Lackner, Andelko Hrzenjak, Thomas Eichmann, Harald C. Köfeler, Katharina Leithner

**Affiliations:** 1 Division of Pulmonology, Department of Internal Medicine, Medical University of Graz, Auenbruggerplatz 15, 8036 Graz, Austria; 2 Department of Experimental Oncology, European Institute of Oncology, 20139 Milan, Italy; 3 Department of Biosciences and Medical Biology, Bioanalytical Research Labs, University of Salzburg, 5020 Salzburg, Austria; 4 Division of General, Visceral and Transplant Surgery, Department of Surgery, Medical University of Graz, 8036 Graz, Austria; 5 Core Facility Mass Spectrometry and Lipidomics, ZMF, Medical University of Graz, 8036 Graz, Austria; 6 Ludwig Boltzmann Institute for Lung Vascular Research, 8010 Graz, Austria; 7 BioTechMed-Graz, 8010 Graz, Austria

**Keywords:** Lung cancer, Metabolism, Serine, Glycine, Starvation

## Abstract

Serine and glycine give rise to important building blocks in proliferating cells. Both amino acids are either synthesized de novo or taken up from the extracellular space. In lung cancer, serine synthesis gene expression is variable, yet, expression of the initial enzyme, phosphoglycerate dehydrogenase (PHGDH), was found to be associated with poor prognosis. While the contribution of de novo synthesis to serine pools has been shown to be enhanced by serine starvation, the impact of glucose deprivation, a commonly found condition in solid cancers is poorly understood. Here, we utilized a stable isotopic tracing approach to assess serine and glycine de novo synthesis and uptake in different lung cancer cell lines and normal bronchial epithelial cells in variable serine, glycine, and glucose conditions. Under low glucose supplementation (0.2 mM, 3–5% of normal plasma levels), serine de novo synthesis was maintained or even activated. As previously reported, also gluconeogenesis supplied carbons from glutamine to serine and glycine under these conditions. Unexpectedly, low glucose treatment consistently enhanced serine to glycine conversion, along with an up-regulation of the mitochondrial one-carbon metabolism enzymes, serine hydroxymethyltransferase (SHMT2) and methylenetetrahydrofolate dehydrogenase (MTHFD2). The relative contribution of de novo synthesis greatly increased in low serine/glycine conditions. In bronchial epithelial cells, adaptations occurred in a similar fashion as in cancer cells, but serine synthesis and serine to glycine conversion, as assessed by label enrichments and gene expression levels, were generally lower than in (PHGDH positive) cancer cells. In summary, we found a variable contribution of glucose or non-glucose carbon sources to serine and glycine and a high adaptability of the downstream one-carbon metabolism pathway to variable glucose supply.

**Supplementary Information:**

The online version contains supplementary material available at 10.1186/s40170-024-00337-3.

## Introduction

Tumor cells rewire their metabolism to support tumor initiation, growth and metastasis [1, 2]. The tissue of origin, genetic alterations and perturbed signaling networks are the main cell-intrinsic determinants of cancer cell metabolic rewiring [1, 2]. However, the metabolic phenotype of cancer cells is extensively modulated by nutrient availability [1, 2]. The latter can be greatly diminished in solid cancers, due to the rapid consumption and inadequate supply, limiting the uptake of certain crucial building blocks such as glucose and serine [3, 4]. Glucose levels were found to be reduced in the interstitial fluid of genetically engineered cancers in mice compared to plasma [5], and in tissues from different cancers, including lung cancer compared to normal tissues [6–11]. Serine, a non-essential amino acid that is either taken up or synthesized by cells de novo, is required for the synthesis of proteins and lipids, including sphingolipids and phospholipids [12, 13]. Via conversion to glycine by serine hydroxymethyltransferase (SHMT), serine contributes to the folate cycle, a series of reactions transferring one-carbon units for nucleotide synthesis [14, 15]. In the serine synthesis pathway (SSP), phosphoglycerate dehydrogenase (PHGDH) mediates the conversion of the glycolytic intermediate 3-phosphoglycerate (3-PG) to 3-phosphohydroxypyruvate, phosphoserine aminotransferase (PSAT1) catalyzes the transamination to 3-phosphoserine, and phosphoserine phosphatase (PSPH) generates serine [14, 15]. Enzymes of the SSP, SHMT1 and SHMT2 (cytoplasmic and mitochondrial isoform, respectively), as well as downstream one-carbon metabolism enzymes are overexpressed in numerous cancers compared to the normal tissues (reviewed in [15]). Due to the importance of nucleotide synthesis in proliferating cells, glycine and one-carbon metabolism are tightly linked to the proliferation rate of cancer cells [16].

Lung cancer is the most frequent cause of cancer deaths worldwide [17]. The prognosis is still poor despite novel therapies targeting immune checkpoints and tyrosine kinases [18]. Metabolic vulnerabilities in lung cancer are still incompletely understood [19]. In vivo stable isotopic tracing in lung patients revealed a high uptake of glucose in most tumors, however, also non-glucose carbon sources were utilized and considerable inter- and intrapatient heterogeneity was discovered [20, 21]. In non-small cell lung cancer (NSCLC), the largest group of lung cancers, the expression of SSP enzymes has been reported to be highly heterogeneous in part due to up-regulation by nuclear factor erythroid 2-related factor 2 (NRF2), a target of the frequently mutated Kelch-like ECH-associated protein 1 (KEAP1) [22, 23]. In lung adenocarcinoma, the most frequent subtype of NSCLC, high PHGDH protein [24] and a high SSP enzyme mRNA expression [22, 23] predicted poor overall survival. SHMT2 and methylenetetrahydrofolate dehydrogenase, MTHFD2, key enzymes of the mitochondrial folate cycle, were likewise found to be overexpressed in lung adenocarcinoma and/or associated with poor prognosis [23–25].

Serine and glycine starvation has been found to increase the contribution of the SSP to serine and glycine pools and to activate the SSP [26–31]. Moreover, the sensitivity towards SSP inhibition is enhanced by serine and glycine deprivation [27–32]. However, certain cancers appear not to fully compensate for extracellular serine by the SSP, as a serine- and glycine-free diet, which led to a significant lowering of plasma serine levels, has been shown to reduce tumor growth in some in vivo cancer models [12, 28, 29]. Thus, the functions of the SSP and serine catabolic pathways in cancer cells are diverse and their metabolic role in different cancer cell phenotypic states and microenvironmental conditions are just slowly being unraveled.

It has been shown that gluconeogenesis, in essence the reverse pathway of glycolysis, can be activated in lung cancer cells and other cancer types as an adaptive response to glucose deprivation [33–41], allowing cancer cells to divert glycolytic intermediates into the SSP even in the absence of glucose [35, 37–39]. Still, the interplay of serine synthesis, uptake and catabolism in cancer cells under starvation is poorly understood. Adaptation of cancer cells to nutrient shortage, however, is increasingly viewed as an important contributor to cancer progression and may be different from adaptive responses in normal cells [4]. Thus, we set out to analyse the metabolic adaptive responses of lung cancer cell lines versus normal, primary bronchial epithelial cells to different (patho-) physiological ranges of serine, glycine and glucose.

## Materials and methods

### Cancer cell lines

The human lung cancer cell line A549 was obtained from Cell Lines Service (Eppelheim, Germany). A549 cells were cultured in DMEM/F-12 (Gibco, Waltham, MA, USA) supplemented with 2 mM glutamine (Gibco), 10% fetal bovine serum (FBS, Biowest, Nuaillé, France) and antibiotics (Gibco). The human lung cancer cell lines NCI-H23, NCI-H358, NCI-H441 and NCI-H1299 were purchased from the American Type Culture Collection (ATCC, Manassas, VA, USA). NCI-H460 cells were a kind gift from Martin P. Barr, Institute of Molecular Medicine, St. James’s Hospital and Trinity College Dublin, Dublin, Ireland, to A.H. H23, H358 and H441 cells were cultured in RPMI 1640 (Gibco) supplemented with 2 mM glutamine, 10% FBS and antibiotics. H460 and H1299 cells were cultured in DMEM (Gibco) supplemented with 10 mM glucose, 2 mM glutamine, 10% FBS and antibiotics. Cell line authentication was done for all cell lines by Short Tandem Repeat (STR) analysis using the PowerPlex 16HS System (Promega, Madison, WI, USA).

### Bronchial epithelial cells

Primary bronchial epithelial cells (BEC) were isolated from non-utilized human donor lungs, explanted at the Department of Surgery, Medical University of Graz, Austria (P.S.). For BEC isolation, a protocol modified after Fulcher et al. [42] was used. Briefly, proximal to segmental bronchi from explant lungs were cut in approx. 1 × 2 cm pieces, rinsed with PBS and the epithelium was gently scraped. The cells were resuspended in DMEM/F12 medium supplemented with 20% FBS, antibiotics and antimycotic (Fungizone, 1:100; Gibco, Waltham, MA) and the suspension was transferred onto a cell culture dish coated with collagen type I (rat-tail collagen, 3 mg/ml, Gibco, diluted 1:100 in PBS). After cell attachment, the medium was replaced with bronchial epithelial cell growth medium (BEGM, CC-3170, Lonza, Basel, Switzerland) containing growth factors. Cells were grown under standard conditions at 37°C, 5% CO_2_ and 95% humidity and used in low passages (≤ 5). BEC were 99.9% cytokeratin positive upon staining with a pan-cytokeratin, mouse monoclonal antibody (Clone: MNF116, 1:100, Invitrogen, Thermo Fisher, Waltham, MA, USA).

### Starvation treatments

Cells were plated at 21,000 cells per cm^2^ in 6-well plates in normal growth media. After 24 h cells were washed twice with phosphate buffered saline (PBS) and treated with the respective starvation or non-starvation media. For the initial screening, DMEM or SILAC RPMI 1640 medium (Gibco) lacking glucose and glutamine were supplemented with 10 mM or 0.2 mM glucose (Sigma-Aldrich, St. Louis, MO, USA), 2 mM glutamine and 0% or 10% dialyzed FBS (Gibco). SILAC RPMI was additionally supplemented with arginine and lysine (Sigma-Aldrich) at concentrations present in normal RPMI 1640. For serine/glycine and glucose starvation, DMEM without glucose, glutamine, serine and glycine, containing standard cystine (Biomol, Hamburg, Germany) was supplemented with serine, glycine and glucose (Sigma-Aldrich) at the indicated concentrations. Glutamine was added at a concentration of 2 mM. In some experiments, custom-made DMEM medium lacking glucose, glutamine, serine, glycine, cystine, cysteine, pyruvate and phenol red (Cell Culture Technologies, Gravesano, Switzerland) was used. For the supplementation with different levels of cystine, a 100 mM stock in 1 M HCl was freshly prepared. During the course of experiments media were refreshed every 24 h, unless indicated otherwise.

### Viability assay and cell counts

For the assessment of cell viability, cells were plated into 12-well plates and treated exactly as described above for 36 h. Medium replacement after 24 h was omitted in order to allow harvesting of all dead/floating cells. After collection of floating cells and trypsinization of attached cells, cells were centrifuged and the pellet was resuspended in CellTrace™ Calcein Red–Orange dye 1:5000 in PBS and incubated for 20 min at 37 °C according to the manufacturer’s instructions. The percentage of Calcein-positive (viable) cells was measured with flow cytometry (CytoFlex, Beckman Coulter, Barea, California, USA). Viable cell counts were determined using the CASY Cell counter (OMNI Life Science, Bremen, Germany).

### Stable isotopic tracing and gas-chromatography/mass spectrometry (GC–MS)

Methods for stable isotopic tracing and GC–MS are explained in detail in Supporting information. Briefly, cells were pretreated with the respective media for 24 h and ^13^C tracing was performed for additional 24 h. In case of parallel labeling with different tracers (e.g. ^13^C_6_-glucose and ^13^C_5_-glutamine) the tracers were added in the same experiment in parallel wells. GC–MS was essentially performed as described [37, 43]. El-Maven software [44] was used for peak quantification and correction for natural abundance was performed with Isocor [45].

### Statistics

Data were compiled and analyzed with the software package SPSS, version 28.0 (Chicago, IL) or with Prism 7 (Graph Pad, Boston, MA). Group differences were calculated using two-sided Student´s t-test, one group Student’s t-test, One-Way or Two-way ANOVA with post-hoc analysis or Mann Whitney-U test, as appropriate. Univariate survival analysis was performed using Log-rank test and the median value of expression was used as the cutoff. Overall survival was defined as time from surgery to death. Patients who survived less than 30 days after surgery were excluded from the analysis. The Cox proportional hazards model was used for multivariate survival analysis. *P*-values smaller than 0.05 were considered significant.

## Results

### Expression of serine synthesis and catabolism enzymes in lung cancer samples and cell lines

**Fig. 1:**
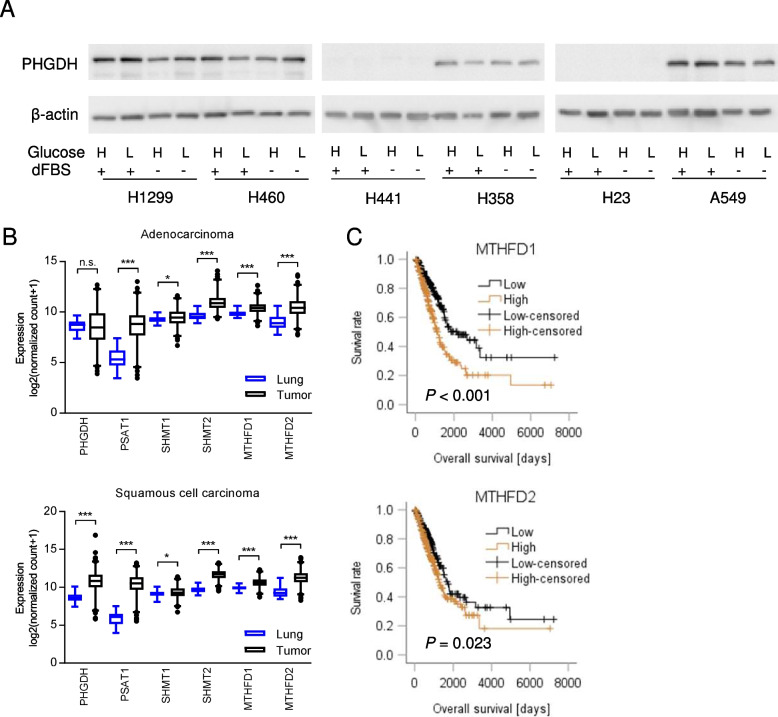
Serine synthesis pathway and one-carbon metabolism enzymes are overexpressed in lung cancer compared to normal lung. **A** PHGDH expression in lung cancer cell lines treated with 10 mM (high, H) or 0.2 mM (low, L) glucose with or without dialyzed serum (dFBS). Beta-actin was used as a loading control. **B** Gene expression in lung adenocarcinoma (LUAD, *n* = 514) or lung squamous cell carcinoma (LUSC, *n* = 502) and non-involved lung (*n* = 59 and *n* = 51, respectively) obtained from the publicly available TCGA dataset via the XENA database (https://xenabrowser.net/). Group comparisons were performed by Mann–Whitney U tests. * *P* < 0.05; *** *P* < 0.001; n.s., not significant. **C** Overall survival in lung adenocarcinoma patients (*n* = 491) from the TCGA dataset expressing low or high levels of MTHFD1/2. Median values were used as a cut-off. Survival analysis was performed using Log-rank test

First, we assessed PHGDH expression in NSCLC cells treated with different concentrations of glucose, a high physiological plasma level of 10 mM (high glucose) or a non-zero level of 0.2 mM (low glucose) in serum-containing or serum-free media. We identified cell lines with high or low/absent PHGDH expression (Fig. 1A). Four lung cancer cell lines, A549, H1299, H460 and H358 showed robust PHGDH expression, and two of them, A549 and H460, in fact harbour a KEAP1 mutation [46]. Two of six NSCLC cell lines, H441 and H23, exhibited undetectable levels of PHGDH protein. These two, but not the other cell lines, show a mutation in TP53 [47]. Of note, p53 (TP53) has been previously shown to suppress SSP enzyme expression and sensitivity towards SSP inhibition [28].

In the TCGA datasets of the two main subtypes of NSCLC, adenocarcinoma (LUAD) and squamous cell carcinoma (LUSC), PSAT1, SHMT1, SHMT2, MTHFD1 and MTHFD2 were significantly overexpressed compared to normal lung tissue (Fig. 1B). PHGDH was up-regulated only in LUSC (Fig. 1B). MTHFD1 and MTHFD2, but not SSP genes or SHMT1/2, were significantly associated with worse overall survival in LUAD patients from the TCGA cohort (Fig. 1C, Supplementary Figure S1), MTHFD1, the cytoplasmic isoform, was even an independent prognostic factor in LUAD patients in multivariate survival analysis including also the tumor stage (Table 1). In LUSC patients, MTHFD1 or MTHFD2 were not linked to prognosis (Supplementary Figure S1). We noted a significant association of SSP genes as well as SHMT2 and MTHFD2 with the expression of the asparagine synthase (ASNS), an activating transcription factor 4 (ATF4) regulated marker for amino acid and glucose deprivation [48] in both lung cancer subtypes (Supplementary Table S1). Together, these and the published data show that serine synthesis and catabolism enzymes are overexpressed in lung cancer, that their expression correlates with a metabolic stress marker and that MTHFD1/2 mRNA expression is linked to poor survival in adenocarcinoma.

**Table 1:** Multivariate analysis of MTHFD1 expression and overall survival in lung adenocarcinoma patients (TCGA dataset)

**Factor**	**Relative risk**	**95% CI**	***P***
Age at initial diagnosis	1.004	0.988–1.020	0.63
Gender	1.003	0.737–1.364	0.99
Stage			**< 0.001**
IB vs. IA	1.185	0.718–1.957	0.51
IIA vs. IA	**3.232**	1.813–5.761	**< 0.001**
IIB vs. IA	**2.016**	1.187–3.425	**0.009**
IIIA vs. IA	**3.441**	2.080–5.694	**< 0.001**
IIIB vs. IA	**2.698**	1.105–6.587	**0.03**
IV vs. IA	**3.939**	2.092–7.416	**< 0.001**
MTHFD1 (high vs. low)	**1.713**		**< 0.001**

**Fig. 2:**
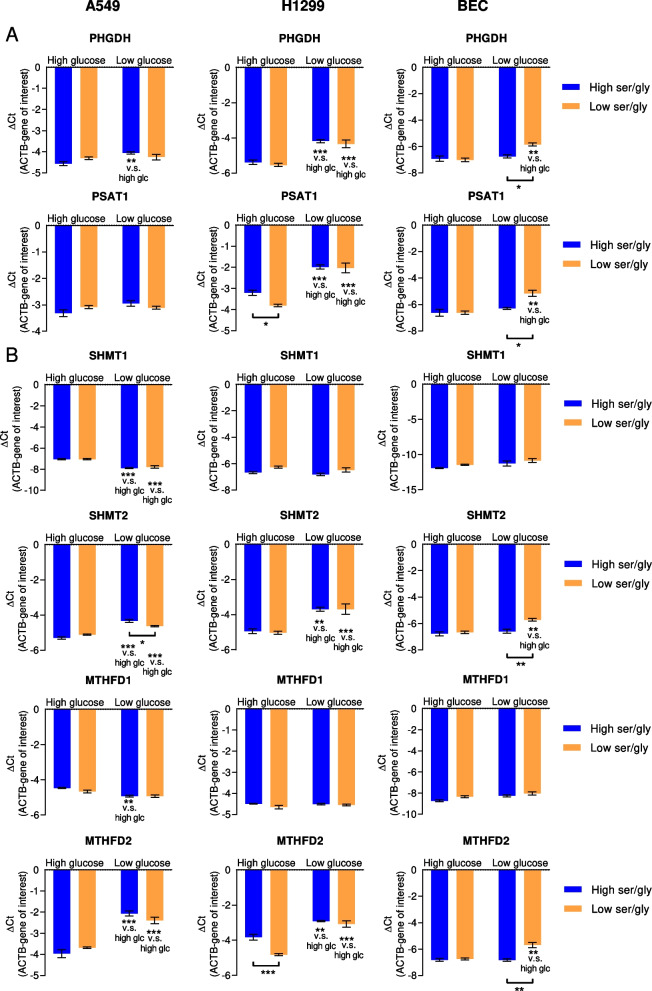
Expression of serine synthesis pathway and one-carbon metabolism genes under serine/glycine or glucose deprivation. Normal BEC or cancer cells were treated with media containing different concentrations of glucose in combination with high/low levels of serine (150 µM/15 µM) and glycine (300 µM/30 µM) for 48 h. **A** Expression of SSP genes and **B** serine and glycine catabolic genes was assessed by quantitative PCR. Results are mean ± SEM from four (A549 and H1299) or three (BEC) experiments. Group comparisons were performed by Two-way ANOVA with Tukey post-hoc analysis; *P < 0.05; **P < 0.01; ***P < 0.001; n.s., not significant; ser/gly, serine/glycine; PHGDH, phosphoglycerate dehydrogenase; PSAT1, phosphoserine aminotransferase; SHMT1, serine hydroxymethyltransferase cytoplasmic isoform; SHMT2, serine hydroxymethyltransferase mitochondrial isoform; MTHFD1, methylenetetrahydrofolate dehydrogenase cytoplasmic isoform; MTHFD2, methylenetetrahydrofolate dehydrogenase mitochondrial isoform

For our starvation treatments, we utilized serum-free medium (DMEM) containing plasma (“high”) levels of serine (150 µM) and glycine (300 µM) [49], or 10% of these concentrations (“low”) and combined these with high (10 mM) or low (0.2 mM) glucose conditions. Of note, fasting plasma glucose levels in normal, non-diabetic individuals are approximately 4–6 mM and may rise up to 8 mM postprandially [50]. In the tumor interstitial fluid of murine pancreatic cancer, glucose concentrations were on average half the level of plasma [5], and in the tumor interstitial fluid of different rat tumors collected by a chamber system, glucose levels were frequently at or below the detection limit [51]. Thus, we chose to select a clearly reduced, non-zero level of glucose (0.2 mM) to mimic glucose deprivation in the solid tumor microenvironment and the treatment media were replaced every 24 h. Since glycine can be converted to serine in the reverse SHMT reaction [52], serine and glycine were reduced in parallel. Normal BEC cells and the cancer cell lines A549 and H1299 readily tolerated the treatment with these media, with only insignificant reductions of viable cell numbers and no increase in cell death (Supplementary Figure S2A,B). In contrast, H460 cells, showed clearly reduced viability under low glucose (Supplementary Figure S2A,B). Interestingly, H460 cells harbor a phosphatidylinositol 3-kinase mutation which is known to hyperactivate glycolysis via Akt serine/threonine-protein kinase [4]. Low exogenous levels of serine, glycine or glucose led to cell-line dependent changes in gene expression levels of SSP enzymes (Fig. 2A). In H1299 cells, low glucose treatment led to an increase in PHGDH and PSAT1 mRNA irrespective of serine/glycine concentrations, while low serine treatment decreased PSAT1 mRNA, however, only in high glucose conditions. Likewise, in A549 cells, low versus high glucose treatment significantly elevated PHGDH expression, but the increase was limited to high serine/glycine conditions. In BEC, PHGDH and PSAT1 expression were increased by a combination of low glucose and low serine/glycine treatment (Fig. 2A). On the protein level, H1299 cells showed a trend for increased PSAT1 protein levels under low glucose conditions compared to high glucose conditions, however, the changes were not significant (Supplementary Figure S3C). SSP enzyme expression was not significantly modulated by the respective treatments in A549 cells or BEC (Supplementary Figure S3B,D). Overall, the increase of SSP gene expression by glucose starvation was not clearly reflected by higher protein levels. Interestingly, the mitochondrial serine and glycine catabolic enzymes SHMT2 and MTHFD2 showed robust elevations in low glucose versus high glucose treatment in the cancer cells and in BEC on the mRNA levels, but in the latter the increase only occurred in combination with low serine/glycine availability (Fig. 2B).

### The contribution of glycolysis to serine pools is increased in low serine/glycine conditions and partly elevated in low glucose conditions

**Fig. 3:**
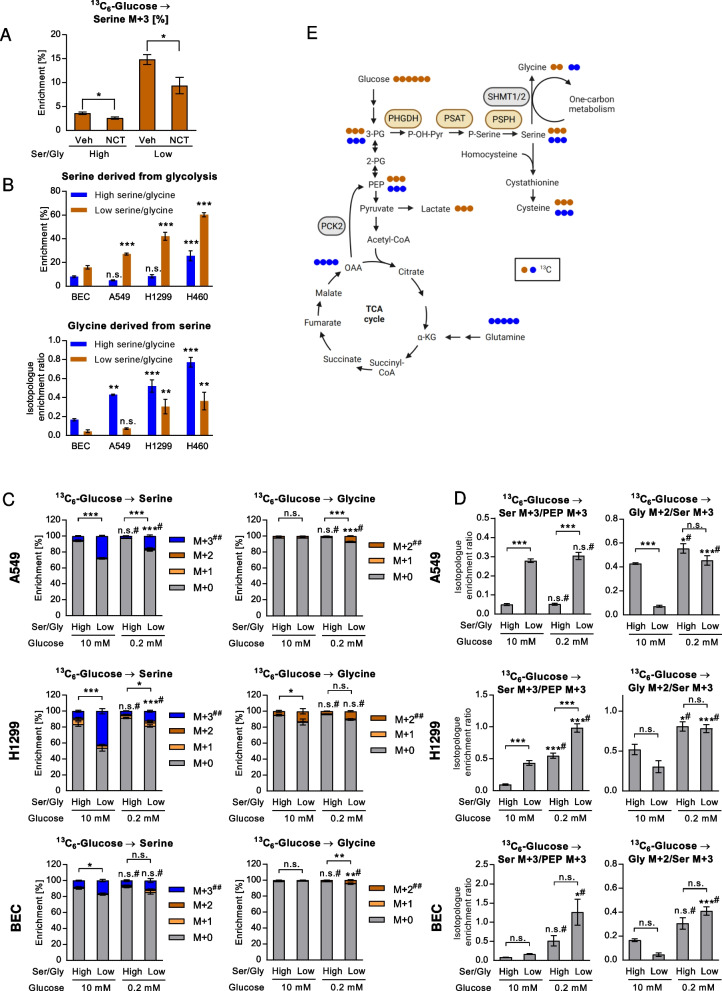
Serine synthesis from glucose and the impact of exogenous serine/glycine and glucose levels. Uniformly labeled [^13^C_6_]-glucose was administered to normal BEC or cancer cells at two different concentrations, high (10 mM) or low (0.2 mM), in combination with high/low levels of serine (150 µM/15 µM) and glycine (300 µM/30 µM) and metabolites were quantified. **A** Label enrichment (% of total) in A549 cells from ^13^C_6_-glucose after treatment with NCT-503 (NCT, 10 µM) or vehicle (Veh). **B** Serine labeling from ^13^C_6_-glucose and relative glycine labeling from serine in normal and cancer cells. Label enrichments in the cancer cells were compared to BEC. **C** Serine and glycine isotopologue enrichments. M + 0 denotes unlabeled metabolites and M + 1, M + 2 and M + 3 contain one, two, or three ^13^C. Statistical analysis was performed on relative enrichment of the most informative isotopologue (##). **D** Enrichment ratios of (fully labeled) serine M + 3 normalized to (fully labeled) phosphoenolpyruvate (PEP) M + 3 and (fully labeled) glycine M + 2 normalized to serine M + 3. **A**-**D** Results are mean ± SEM from four (A549 and H1299) or three (BEC) experiments. Group comparisons were performed by Two-way ANOVA with Tukey (A,C-D) or Dunnett (B) post-hoc analysis; *P < 0.05; **P < 0.01; ***P < 0.001; n.s., not significant; #, versus high glucose conditions; Ser/Gly, serine/glycine; **E** Metabolic pathway for the conversion of glucose or glutamine to serine/glycine and cysteine. Filled circles represent ^13^C. TCA cycle, tricarboxylic acid cycle; PEP, phosphoenolpyruvate; PCK2, phosphoenolpyruvate carboxykinase mitochondrial isoform

In order to analyze the impact of exogenous serine/glycine on SSP activity under different glucose concentrations, we utilized uniformly ^13^C-labeled (^13^C_6_-) glucose and measured ^13^C enrichment in serine and the downstream product glycine. Serine derived from uniformly ^13^C_6_-glucose is labeled at all three carbon positions (M + 3) and converted to glycine carrying two ^13^C (M + 2). The labeling patterns are depicted in Fig. 3E. As expected, blocking of the SSP using the pharmacological PHGDH inhibitor NCT-503 [53] reduced the relative contributions of ^13^C_6_-glucose to serine (Fig. 3A). We pre-treated cells in high or low serine and glycine in media containing high (10 mM) or low (0.2 mM) glucose for 24 h. Then we replaced unlabeled by labeled glucose at the same concentrations and ^13^C labeling continued for 24 h. In low serine/glycine medium, the relative enrichment of uniformly (M + 3) labeled (de novo synthesis derived) serine was clearly increased compared to high serine/glycine conditions in all investigated cancer cell lines (A549, H1299 and H460) as well as in normal BEC under high glucose conditions (Fig. 3B,C, Supplementary Figure S4B). This finding is similar to the previously published reports on cancer cells. H460 cells were treated only with high glucose media due to their sensitivity to glucose withdrawal.

The ^13^C enrichment in glycine from ^13^C_6_-glucose was inconsistently changed. In high glucose conditions, only H1299 cells showed an increased glycine labeling in serum/glycine deprivation (Fig. 3C). However, in low glucose conditions all cells showed enhanced glycine labeling under low serine/glycine supplementation. (Fig. 3C). Relative isotopic labeling may be influenced by the metabolite pool sizes, especially if isotopic steady state is not achieved [54]. In fact, total intracellular serine and glycine pools were consistently reduced in low exogenous serine/glycine conditions in all cell lines, mainly due to a reduction of unlabeled (M + 0) serine and glycine (Supplementary Figure S4A), similar to previous reports [26–28, 52]. Thus, low pool sizes may greatly contribute to the enhanced contribution of glucose to serine in low serine/glycine conditions. Changes in serine and glycine labeling may also result from changes in precursor labeling. The serine precursor 3-PG is in exchange with phosphoenolpyruvate (PEP) via the bidirectional enzymes phosphoglycerate mutase and enolase. Assessing the contribution of glycolysis to serine pools by determining the ratio of serine M + 3 and PEP M + 3, we found that serine production from glycolysis was likewise increased in low serine/glycine conditions (Fig. 3D). M + 3 labeling of PEP was quite similar in low and high serine/glycine conditions (Supplementary Figure S5). However, labelling of PEP and the downstream glycolytic intermediates pyruvate and lactate was clearly reduced in low glucose compared to high glucose conditions (Supplementary Figure S5), yet in A549 cells almost half of PEP pools were fully ^13^C labeled from ^13^C_6_-glucose despite the low concentration of the tracer. Supplementary Figure S6 shows the respective metabolite abundance data. Thus, changes in serine/glycine labeling in low and high serine/glycine conditions did not occur due to different precursor label enrichments. Labeled serine and glycine were also exported to the media (Supplementary Figure S7A-C), in line with published data [22], indicating an exchange of intracellular and extracellular serine and glycine.

**Fig. 4:**
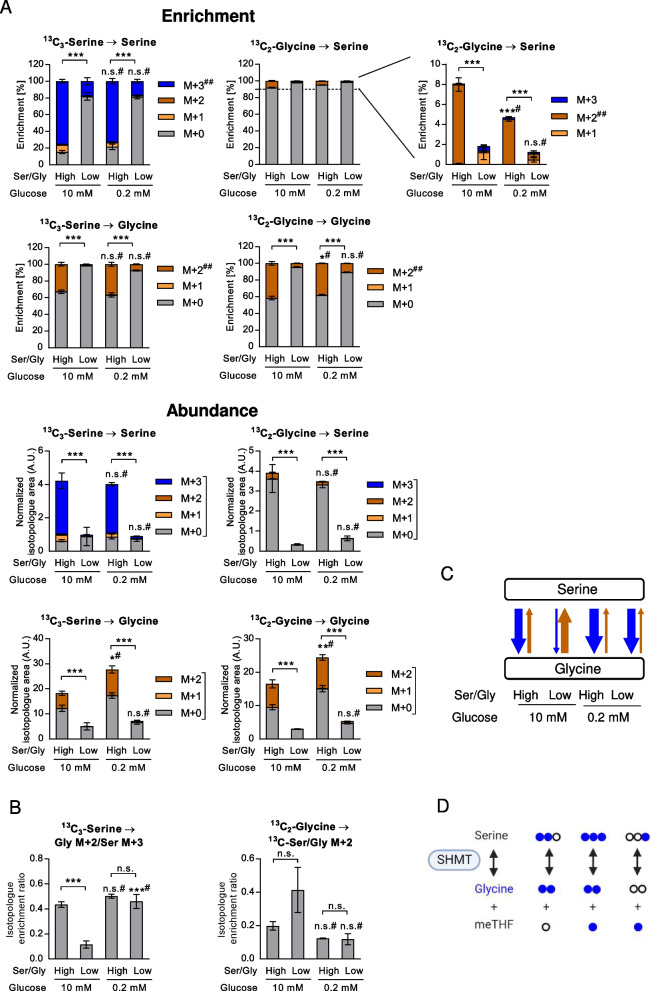
Intracellular serine and glycine derived from exogenous serine and glycine supplied at high or low concentrations. Uniformly ^13^C labeled serine or glycine were used in A549 cells at high or low concentrations as tracers in media containing high or low glucose. **A** Enrichment (top) and total abundance normalized to protein (bottom) of serine and glycine. For the statistical analysis of enrichment the most informative isotopologue (##) was used. For abundance data, statistical analysis was performed on total metabolite abundance. **B** Enrichment ratios of (fully labeled) glycine M + 2 fractions normalized to (fully labeled) serine M + 3 and labeled serine (sum of M + 1, M + 2 and M + 3) fractions normalized to (fully labeled) glycine M + 2, respectively. **A**-**B** Results are shown as mean ± SEM from four independent experiments. Group comparisons were performed using Two-way ANOVA with Tukey post-hoc analysis. *P < 0.05; **P < 0.01; ***P < 0.001; n.s., not significant; # versus high glucose conditions. Ser/Gly, serine/glycine; **C** Summary of contributions from serine to glycine and vice versa, based on ratios shown in **B**. **D** Serine and glycine interconversion by SHMT. Filled circles represent ^13^C. meTHF; 5,10-methylene tetrahydrofolate

In order to address the question, whether modifications of exogenous serine/glycine had an impact on serine and glycine uptake, we utilized uniformly labeled [^13^C_3_]-serine and [^13^C_2_]-glycine in A549 cells (Fig. 4A). While approximately 75% of serine and approximately 42% of glycine were derived from the import of exogenous serine and glycine, respectively, at plasma concentrations of the two amino acids, this contribution was clearly reduced in low serine/glycine conditions (17% and 4.4%). This decrease also greatly affected total serine and glycine abundance (Fig. 4). Together, these data show that the contribution of the SSP to serine pools increases with lower exogenous serine and glycine supply and uptake. Importantly, glucose conversion to serine and serine conversion to glycine, assessed by calculating the ratio of glycine M + 2 fractional enrichment normalized to serine M + 3 derived from ^13^C_6_-glucose, both were significantly higher in all the PHGDH positive cancer cell lines examined compared to the normal BEC (Fig. 3D). This increased activity occurred parallel to enhanced expression of SSP and one-carbon metabolism genes in the cancer cells compared to BEC (Supplementary Figure S3A).

### Low glucose conditions activate the SSP from glucose in a cell line-dependent manner

Low glucose treatment clearly reduced the abundance of lactate in cells (Supplementary Figure S6) as well as the release from the cells (Supplementary Figure S7). Under low ^13^C_6_-glucose conditions, a maximum of half of the pools of PEP were fully ^13^C labeled (M + 3), compared to nearly 100% labeling in high glucose conditions (Supplementary Figure S5). Unexpectedly, when we normalized serine labeling (M + 3 enrichment) to the labeling of PEP, we found that low glucose conditions consistently activated serine de novo synthesis from glucose in H1299 cells and in BEC (Fig. 3D). In A549 cells, normalized serine labeling was similar in high or low glucose (Fig. 3D). This enhancement of SSP under low glucose was not caused by differences in serine pool sizes (Supplementary Figure S4A). Thus, the SSP is unexpectedly maintained (A549) or even activated (H1299 and BEC) under low glucose availability.

### Low serine/glycine conditions inhibit, while low glucose conditions enhance glycine formation from serine

When we assessed glycine formation from serine, reflected by relative glycine M + 2 labeling from serine M + 3, we found an unexpected decrease by low serine/glycine conditions in a high glucose environment (Fig. 3D and Supplementary Figure S4B). This change was observed in all cancer cell lines as well as in normal BEC, yet it reached significance only in A549 and H460 cells (Fig. 3D and Supplementary Figure S4B). The decrease of normalized glycine labeling in low serine/glycine treatment was readily observed also with serine as a tracer (Fig. 4B). Interestingly, the decline of glycine formation from serine was completely blunted in a low glucose environment (Figs. 3D, 4B and Supplementary Figure S4B). In fact, the relative labeling in glycine from serine significantly increased in low glucose in all cancer cell lines and BEC (Fig. 3D). The reverse pathway, glycine to serine conversion via the reverse SHMT reaction, leads to different labeling pattern depending on the labeling of glycine and of the one-carbon unit attached to the cofactor tetrahydrofolate (5,10-methylene-THF) (Fig. 4D). Glycine to serine conversion was found in all conditions. When fractional serine labeling was normalized to glycine labeling, an increase was found in high glucose/low serine/glycine conditions, however, the increase did not reach significance (Fig. 4B). Figure 4C represents the different contributions from serine to glycine and vice versa.

### Changes of glycolytic intermediates under serine/glycine or glucose starvation and a possible allosteric modulation

**Fig. 5:**
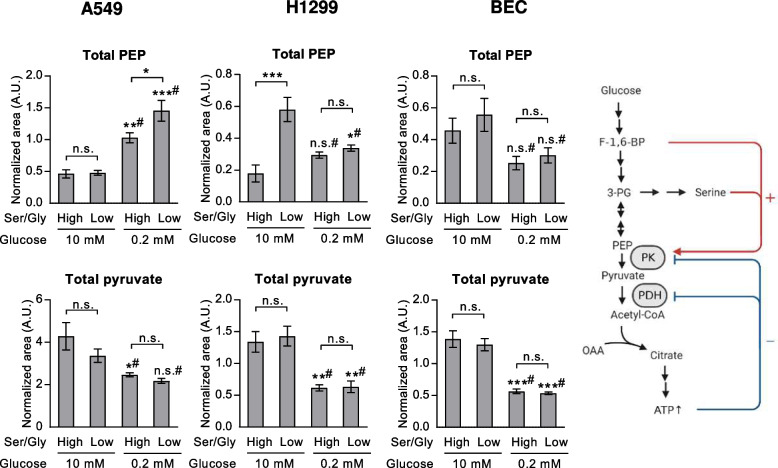
Lower glycolytic intermediates in high or low serine/glycine or glucose. Total abundance (normalized to protein and internal standard) of phosphoenolpyruvate (PEP) and pyruvate from experiments shown in Fig. 3. Results are mean ± SEM from four (A549 and H1299) or three (BEC) experiments. Group comparisons were performed by Two-way ANOVA with Tukey post-hoc analysis; *P < 0.05; **P < 0.01; ***P < 0.001; n.s., not significant; # versus high glucose conditions. Ser/Gly, serine/glycine; Right: Known allosteric modulators of lower glycolysis**.** PK, pyruvate kinase; PDH, pyruvate dehydrogenase

The enhanced expression of PSAT and PHGDH mRNA under low glucose and low serine, which likely occurs due to activation of the ATF4 pathway [22, 55], may contribute to an enhanced SSP under these conditions. However, allosteric mechanisms may also play a role. Serine allosterically activates PKM2, the isoform of pyruvate kinase often present in tumor cells [26, 56]. A reduction of PKM2 activity under serine deprivation was shown to increase 3-PG availability for serine synthesis, activating the SSP [26, 56]. Likewise, fructose-1,6-biphosphate (F-1,6-BP) is a well-established allosteric activator of pyruvate kinase, resulting in feed-forward activation, while ATP is an inhibitor of pyruvate kinase [57] (scheme depicted in Fig. 5). In our experiments, low serine/glycine levels indeed led to enhanced total PEP, but only in glucose-starved A549 cells (Fig. 5), similar to previously published reports [26, 28]. Low glucose versus high glucose treatment led to an increase of total levels of PEP in A549 cells, while by trend a decrease of PEP occurred in BEC cells: On the other hand, low glucose treatment reduced levels of pyruvate in all cell types (Fig. 5). The changes of PEP and pyruvate in opposite directions in A549 cells may reflect modulation of pyruvate kinase activity, which might contribute to regulation of the SSP pathway activity.

### Gluconeogenesis from glutamine contributes to PEP, serine and glycine to a similar extent as glucose in low glucose conditions

**Fig. 6:**
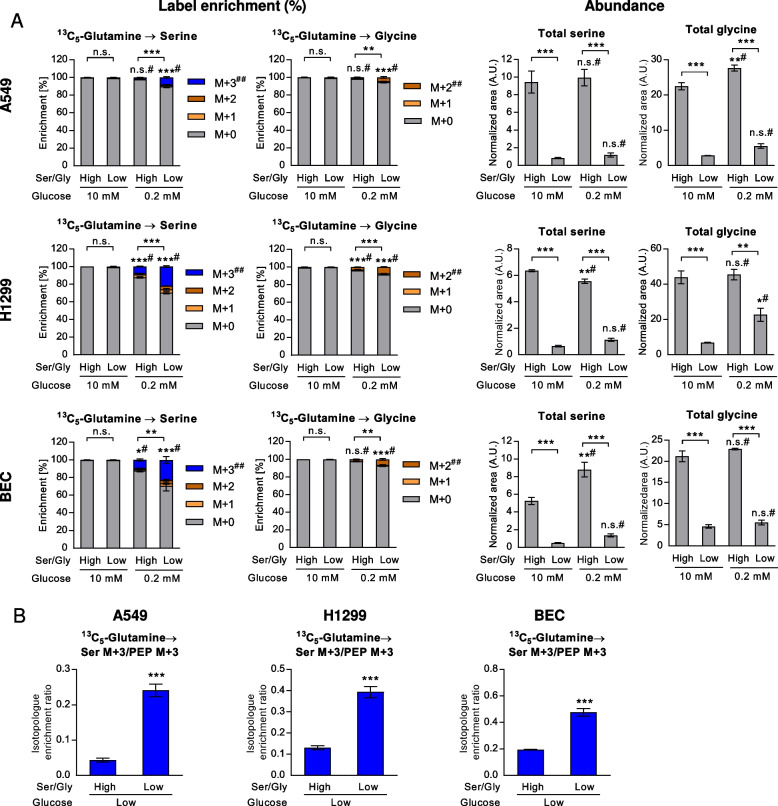
Serine and glycine de novo synthesis from gluconeogenesis. ^13^C_5_-glutamine was administered to normal BEC or cancer cells in high or low glucose and high or low serine/glycine conditions. **A** Label enrichment (left) and total abundance normalized to protein and internal standard (right) are shown as mean ± SEM from four (A549 and H1299) or three (BEC) experiments. Statistical analysis was done for the most informative isotopologue (##). Group comparisons were performed by Two-way ANOVA with Tukey post-hoc analysis; **B** Relative label enrichment in serine (M + 3) normalized to M + 3 labeling of the precursor PEP, reflecting serine de novo synthesis from gluconeogenesis. Group comparisons were performed using Students’s t-test. *P < 0.05; **P < 0.01; ***P < 0.001; # versus high glucose. Ser/Gly, serine/glycine

Having found that serine synthesis continues and is even partly increased in low glucose conditions, we were interested in the contribution of the reverse pathway, gluconeogenesis, to serine synthesis in normal and cancer cells. Similar to previous reports in cancer cells, all the cells converted ^13^C_5_-glutamine via the TCA cycle intermediates and oxaloacetate to PEP, but only under low glucose, not in high glucose conditions (Supplementary Figure S8; labeling scheme shown in Fig. 3E). We and others have previously shown that the mitochondrial isoform of phosphoenolpyruvate carboxykinase (PCK2) is the key enzyme in lung cancer cells mediating the conversion of oxaloacetate to PEP in the (truncated) gluconeogenesis pathway [33, 35, 36]. Accordingly, serine and glycine synthesis were also fueled by gluconeogenesis in low glucose conditions in A549 cells, H1299 cells and BEC (Fig. 6A-B). A clear increase of glutamine contribution to serine and glycine was again found in low serine/glycine conditions (Fig. 6A, left), parallel to a decrease in pool size (Fig. 6A, right), also after normalization to PEP M + 3 labeling (Fig. 6B). The respective label enrichments and abundances of the precursor PEP, the TCA cycle and glycolytic metabolites are displayed in Supplementary Figure S8 and Supplementary Figure S9, respectively. We addressed the conversion of serine to cysteine via the transsulfuration pathway, which has been shown to play an important role in some cancers [58], especially under cysteine limitation [59]. Moreover, we assessed the impact of exogenous cystine on the SSP, since the uptake of high levels of cystine may induce disulfide (redox) stress [60] and potentially modulates NRF2 signaling. Cell culture media usually contain the oxidized form, cystine, however, cystine is reduced to cysteine intracellularly [60]. Cystine supplementation (either plasma levels, 100 µM, or low cystine, 10 µM) did not modulate the SSP from ^13^C_5_-glutamine in A549 cells in any of the nutritional conditions (Supplementary Figure S10).

**Fig. 7:**
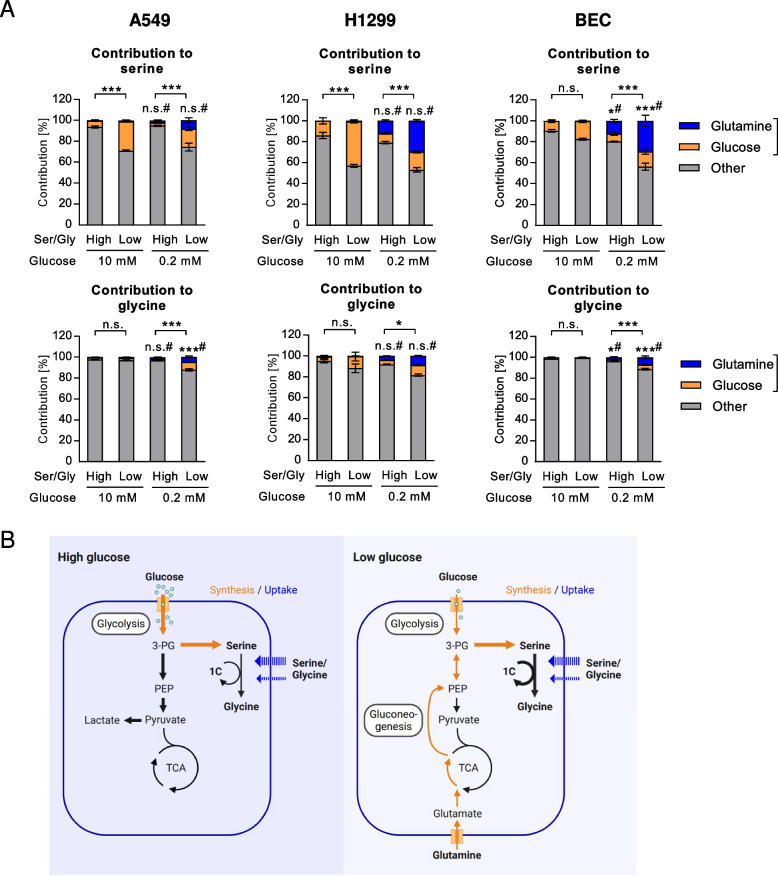
Contribution of glycolysis and gluconeogenesis to serine and glycine. **A** Comparison of label enrichments from ^13^C_6_-glucose or ^13^C_5_-glutamine in pools of serine and glycine. Results are shown as mean ± SEM from four (A549 and H1299) or three (BEC) experiments. The sum of all labeled isotopologues was used for group comparisons using Two-way ANOVA with Tukey post-hoc analysis; *P < 0.05; **P < 0.01; ***P < 0.001; n.s., not significant; # versus high glucose conditions. Ser/Gly, serine/glycine. **B** Model depicting variable contributions of uptake or de novo synthesis from glucose or gluconeogenesis to serine and glycine pools. 1C, one-carbon units; TCA, tricarboxylic acid cycle; PEP, phosphoenolpyruvate; 3-PG, 3-phosphoglycerate

Finally, we compared the contribution of labeled glucose and glutamine, which were used in the same experimental series in parallel wells, to serine and glycine pools. Using this approach potentially an overestimation of labeled metabolite fraction from either source may occur, due to the emergence of partially labeled metabolites, however, their contribution was very low. Interestingly, the contribution from glutamine completely compensated the reduced but still present contribution from glucose in the cancer cells (Fig. 7A). In BEC, however, low glucose conditions led to an overall enhancement of nutrient carbon contribution to serine (Fig. 7A). Glycine labeling increased in combined starvation treatments in all cell types (Fig. 7A). Together, the results of this study show that glycolysis and gluconeogenesis both contribute to the SSP in low glucose, with a marked increase under low exogenous serine/glycine supply (model in Fig. 7B).

## Discussion

Under nutrient shortage, healthy organisms balance the utilization of carbon sources between biomass accumulation for growth (nucleotide, protein and lipid synthesis) and energy production [61]. The cell-autonomous redistribution of carbons for biosynthetic needs under glucose or serine/glycine limitation, which we found in this study, is reminiscent of such a homeostatic response and may be critical for the adaptation of cancer but also normal lung cells to their microenvironment. We show that both, glycolysis and gluconeogenesis contribute carbons to serine and glycine synthesis in normal lung and lung cancer cells, when glucose levels are low (0.2 mM). Moreover, we found that serine to glycine conversion is highly modulated by the extracellular availability of serine and glycine and by the level of glucose. The study also confirms the contribution of both, serine de novo synthesis and uptake, to total cellular serine pools and an increase of the contribution of the SSP under serine and glycine starvation. Together, the data show a highly flexible use of nutrient and carbon sources for serine and glycine generation in lung cancer cells but also normal epithelial cells and a cell-line dependent heterogeneity.

One-carbon metabolism enzymes fueled by serine and glycine are consistently overexpressed in cancers. A recent study showed a clear elevation of glycine and one-carbon metabolism metabolites in NSCLC samples compared to matched normal lung tissue besides other metabolic alterations [62]. Antifolates, which inhibit one-carbon transfer within the folate cycle, have been used in cancer therapy for more than 70 years, yet with dose-limiting toxicities [63]. In fact, pemetrexed, a modern antifolate, is routinely used in the treatment of lung cancer in combination therapies [63]. Especially enzymes of the mitochondrial one-carbon metabolism, SHMT2 and MTHFD2 are frequently increased in cancers, however, cancer cells can partly switch to the cytoplasmic isoforms for the generation of methylene-THF and thus the generation of one-carbon units [64]. In our study we found a robust enhancement of one-carbon metabolism enzymes but also the up-stream SSP enzymes in the two main subtypes of lung cancer on the mRNA level and an association of MTHFD1 and MTHFD2 gene expression with poor survival in lung adenocarcinoma, in line with previously published reports [22–25].

While the formation of one-carbon units for nucleotide synthesis, mitochondrial translation initiation and methylation reactions is a constant requirement in cancer cells, the carbon sources fueling these reactions may vary according to the nutritional state of the cell, as shown by the present and previous studies. The modulation of serine de novo synthesis by glucose availability, however, has not yet been rigorously addressed. Here we found a maintained or even increased SSP under low glucose, in lung cancer but also normal lung epithelial cells. While we found accompanying changes of some SSP enzymes on the mRNA level, the respective protein levels were unchanged or only insignificantly altered. Allosteric regulation or the concentrations of substrates/products may play a role, however, the underlying mechanisms warrant further study. The marked serine production from glycolysis under low glucose (3–5% of the normal blood glucose), despite the clear reduction in lactate formation, supports the concept that glycolysis (the Warburg effect) primarily ensures the generation of building blocks in dividing cells and that a surplus of pyruvate is converted to lactate for export [65].

Interestingly, a lower glycine to serine labeling ratio was found under low versus high serine/glycine conditions in high glucose in all cell lines. SHMT mRNA levels were not altered, however, whether SHMT activity is changed is not known. A reduced demand for products of serine and glycine catabolism, including one-carbon units may play a role. Alternatively, labeled glycine may be diluted with unlabeled glycine from internal stores in low glycine conditions, like glutathione, which is present in cells at relatively high amounts.

Glutamine is an important source of carbons and nitrogen in cancer cells [2]. In previous studies we and others showed that glutamine is used as a precursor in (truncated) gluconeogenesis in glucose-deprived cancer cells and diverted for branching pathways including serine formation [35–40]. However, the modulation of gluconeogenesis and the downstream SSP by exogenous serine availability was still unknown. Recently, enhanced dependence on serine de novo synthesis from gluconeogenesis was found in PCK2-silenced cervical carcinoma cells if the medium was lacking serine or glycine but not if serine/glycine was abundant, suggesting a requirement of cancer cells for both, the SSP and PCK2, under serine and glucose limitation [39]. These and our data show that gluconeogenesis supports the SSP in low glucose conditions which might become limiting under low exogenous supply with serine and/or glycine. The observed simultaneous use of glucose and non-glucose precursors via gluconeogenesis raises the question, whether glycolysis and gluconeogenesis pathways converge at the level of 3-PG, the precursor of serine, or whether glycolysis and gluconeogenesis run in parallel, for example in different cell (sub-) populations. Since only bulk analysis of cells has been performed here and since we did not perform any blockade of gluconeogenic/glycolytic flux at different levels in this study, further research is needed to address this question.

We found that serine uptake and SSP act in concert to fuel serine pools, and that serine uptake is highly dependent on exogenous supply. While these interactions were found in both, normal and cancer cells from the lung, serine de novo synthesis and serine to glycine conversion were consistently elevated in the investigated (PHGDH positive) cell lines compared to normal BEC cells. Still, a clear heterogeneity in SSP enzyme expression was found and some lung cancer cell lines appear to have very little SSP enzyme expression and may rather depend on exogenous serine supply, in line with published data [22]. In our study, the enhanced contribution of de novo synthesis from glucose (or glutamine) in low serine/glycine conditions was paralleled by a decreased contribution from exogenous serine and glycine. In a previous study using dual labeling approaches, indeed a higher contribution of glucose-derived serine and glycine to purine carbons over exogenous serine or glycine uptake was found in lung cancer [66]. This study utilized cell lines but also lung cancer and normal lung explants, which more closely mimic the situation in vivo [66]. Thus, the enhanced SSP is clearly utilized for nucleotide synthesis in lung cancer cells. As mentioned, NRF2 plays an important role in fostering the SSP in lung cancer [22]. Recently, a coordinated enhancement of both, the SSP and serine utilization for nucleotide synthesis has been found upon suppression of nuclear glycogen synthase kinase 3 (GSK3) signaling in lung cancer cells using ^13^C tracers [67]. An increased contribution of the SSP was also shown to be mediated by the transcription factor Myc [66]. From its association with SSP enzyme expression and stimulatory effect of serine synthesis from labeled glucose, also transcription factor NKX2–1 was identified as an activator of the SSP in lung cancer cells [68].

In our study, serine to glycine conversion was reduced under low serine and glycine supplementation. However, this reduction was not found in low glucose conditions, which generally led to increased SSP and serine to glycine conversion. We observed an up-regulation of mitochondrial MTHFD2 but not cytoplasmic MTHFD1 in low glucose. However, downstream one-carbon metabolites were not analyzed and no separate analyses of mitochondrial versus cytoplasmic serine/glycine pools were performed in our study. Interestingly, increased serine catabolism to glycine and formate has been reported in galactose versus glucose fed cells, which primarily occurred in the mitochondria [69]. Moreover, glucose starvation increased protein levels of SHMT2 [70]. Increased conversion of serine to glycine in the mitochondria via SHMT2 has been shown to be required for mitochondrial translation and proliferation of cancer cells under low glucose conditions by providing one-carbon units for the generation of mitochondrial formylmethionyl-tRNAs [71]. However, the modulation of serine to glycine conversion by glucose availability and the role of downstream one-carbon metabolism is still incompletely understood and warrants further study.

In line with previous studies, de novo synthesis of serine occurred irrespective of exogenous serine and glycine, although the relative contribution to the serine pool was greatly increased in low serine/glycine conditions [26–30]. A continuous production of serine may ensure a basal level of intracellular serine in an unstable metabolic tumor microenvironment. However, serine synthesis confers other potential advantages besides the production of serine, including the conversion of oxidized to reduced nicotinamide adenine dinucleotide (NADH), or the transamination of glutamate to α-KG [32, 72]. The requirement for serine, glycine, one-carbon units, and α-KG, however, may be highly dependent on the cellular state, e.g. proliferating versus quiescent and the activity of downstream pathways, like the one-carbon metabolism pathway.

As a limitation, exchange of serine and glycine between the cells and the extracellular space was not accounted for in this study. In line with our data, the intracellular pool of serine and glycine has been shown to be in rapid exchange with the (large) extracellular pool via membrane transporters [22]. Consequently, steady state labelling of serine from glucose may not be easily achieved [22] and the contribution of the respective pathways to the cellular pools, as shown in this study, are not equal true pathway fluxes. In fact, serine or glycine may serve as exchange factors for the import of other amino acids or ions via antiporters. However, this possibility was not addressed in this study. As another limitation of the study, the range of actual glucose, serine, glycine and cystine concentrations in the extracellular fluid of lung cancers is still unknown. The core of solid tumors has been reported to show lower levels of serine than the periphery [73]. In the murine brain, a frequent target organ of lung cancer metastasis, the normal serine concentration in interstitial fluid was as low as 10 µM, compared to 110 µM in plasma [74]. This limited serine supply led to PHGDH dependency in brain metastases of breast cancer [75]. Conversely, serine was not depleted in pancreatic cancer tumor interstitial fluid in mice and glycine was even increased compared to normal plasma, which was attributed to local secretion [5]. Thus, microenvironmental availability of serine and glycine might be highly variable depending on the localization and tumor type.

In conclusion, our study shows a remarkable metabolic flexibility of cancer and normal cells in physiological ranges of glucose, serine and glycine and highlights the role of serine metabolism in low glucose conditions. The remarkable contribution of gluconeogenesis to lower glycolysis and serine/glycine synthesis that is found in both, normal and malignant cells, suggests that cancer cells may retain certain metabolic capabilities of the parental cells to cope with starvation conditions, such as low glucose supply. However, further studies are needed to dissect, how microenvironmental conditions affect metabolic dependencies of cancers with different basal serine synthesis and one-carbon metabolism activation states versus normal cells.

### Supplementary Information

**Additional file 1. **

**Additional file 2: Supplementary Figure S1.** Overall survival in lung adenocarcinoma (n=491) and squamous cell carcinoma patients (*n*=471) from the TCGA dataset expressing low or high levels of SSP or one-carbon metabolism enzymes. Median values were used as a cut-off. Survival analysis was performed using Log-rank test. **Supplementary Figure S2.** Cell counts and viability under starvation conditions. A Viable cell numbers and B the percentage of viable (calcein positive) cells after treatment with the respective starvation media for 36 hours. Results are mean +/- SEM from four independent experiments. Group comparisons were performed by Two-way ANOVA with Tukey post-hoc analysis. B As a positive cell death control, cells treated with serum-free medium lacking glucose, glutamine, serine and glycine was included. Viability was compared to the respective 10 mM glucose, high serine/glycine conditions using One-way ANOVA with Dunnett post-hoc analysis.*P < 0.05; **P < 0.01; ***P < 0.001; n.s., not significant; # versus high glucose; Ser/Gly, serine/glycine. **Supplementary Figure S3.** SSP gene expression in lung cancer and normal lung epithelial cells under starvation treatments. A mRNA levels of SSP and one-carbon metabolism genes in two PHGDH-positive cell lines, A549 and H1299 and normal bronchial epithelial cells (BEC) treated in medium containing high glucose and serine/glycine levels for 48 hours. Results are shown as mean +/- SEM from four (A549, H1299) or three (BEC) independent experiments; Group comparisons were performed by One-way ANOVA with Dunnett post-hoc analysis versus BEC for each gene.***P < 0.001. B-D Representative PHGDH and PSAT1 immunoblots and quantifications of blots from four (A549) or three (H1299 cells or BEC cells) experiments cultured in medium containing high (H) or low (L) serine/glycine, and high (H) or low (L) glucose for 48 2 hours (with medium replacement after 24 hours), with or without variations in cystine (high or low, only A549). β-actin was used as a loading control. Data were normalized to β-actin and to nonstarved cells. Group comparisons were performed by two-sided Student’s t-test or one-group Student’s t-test as applicable. **Supplementary Figure S4.** Serine and glycine abundance in starvation and non-starvation media and serine and glycine enrichment from 13C6-glucose in H460 cells. 13C6-glucose was administered in high glucose medium containing high or low serine/glycine concentrations for 24 hours. A Serine and glycine isotopologue abundance normalized to total protein and internal standard corresponding to Fig. 3. B Serine and glycine enrichment in H460 cells. Fractional enrichments and normalized fractional enrichments are shown. Results are mean +/- SEM from three independent experiments. Group comparisons were performed by A Two-way ANOVA with Tukey post-hoc analysis on total abundance or B two-sided Student’s t-test. *P < 0.05; **P < 0.01;***P < 0.001; n.s., not significant; Ser/Gly, serine/glycine. **Supplementary Figure S5.** Isotopologue enrichment in TCA cycle and glycolysis intermediates derived from 13C6-glucose. 13C6-glucose was administered in a high or low concentration medium containing high or low serine/glycine concentrations for 24 hours. Isotopologue enrichment (% of total) is shown as mean +/- SEM from four (A549, H1299) or three (BEC) independent experiments. The most informative isotopologue (##) was used for group comparisons using Two-Way ANOVA with Tukey post-hoc analysis. *P < 0.05; **P < 0.01; ***P < 0.0, n.s., not significant; # versus high glucose; Ser/Gly, serine/glycine. **Supplementary Figure S6.** Metabolite abundance of TCA cycle and glycolysis intermediates derived from 13C6-glucose. Total metabolite abundance normalized to total protein and internal standard from experiments shown in Supplementary Figure S5. Data are 3 shown as mean +/- SEM from four (A549, H1299) or three (BEC) independent experiments. Group comparisons were performed using Two-Way ANOVA with Tukey post-hoc analysis. *P < 0.05; **P < 0.01; ***P < 0.001; n.s., not significant; # versus high glucose; Ser/Gly, serine/glycine. **Supplementary Figure S7.** Release of lactate, serine and glycine. 13C6-glucose was administered in a high or low concentration medium containing high or low serine/glycine concentrations for 24 hours in A A549 and B H1299 cells. Isotopologue enrichment in medium metabolites, the amount of de novo produced and released (fully 13C-labeled) metabolites normalized to total protein and total metabolite levels in the medium supernatants are shown as mean +/- SEM from four independent experiments. Statistical analysis (Two-Way ANOVA with Tukey post-hoc analysis). For enrichment data, statistical analysis was done for the most informative isotopologue (##). *P < 0.05; **P < 0.01; ***P < 0.001; n.s., not significant; # versus high glucose; Ser/Gly, serine/glycine. **Supplementary Figure S8.** Isotopologue enrichment in TCA cycle and glycolysis intermediates derived from 13C5-glutamine. 13C5-glutamine was administered in a high or low concentration medium containing high or low serine/glycine concentrations for 24 hours. A Serine M+3 to PEP M+3 ratio from 13C5-glutamine, reflecting serine de novo synthesis from gluconeogenesis. Group comparisons were performed using Students’s t-test. ***P < 0.001. B TCA cycle and glycolytic intermediate enrichments. A,B Data are shown as mean +/- SEM from four (A549, H1299) or three (BEC) independent experiments. Ser/Gly, serine/glycine. **Supplementary Figure S9.** Isotopologue abundance of TCA cycle and glycolysis intermediates derived from 13C5-glutamine. Normalized isotopologue abundances from experiments shown in Supplementary Fig. S8. Data are shown as mean +/- SEM from four (A549, H1299) or three (BEC) independent experiments. Group comparisons were performed using Two Way ANOVA with Tukey post-hoc analysis. *P < 0.05; **P < 0.01; ***P < 0.001; n.s., not significant; # versus high glucose; Ser/Gly, serine/glycine. **Supplementary Figure S10.** Impact of exogenous cystine on labeling of cellular intermediates from 13C5-glutamine. 13C5-glutamine was administered in high or low glucose medium containing high or low serine/glycine in combination with high or low cysteine concentrations. Fractional enrichment of gluconeogenesis and SSP intermediates is shown as mean +/- SEM from three independent experiments.

**Additional file 3. **

**Additional file 4. **

## Data Availability

All data are contained within the manuscript.
