# Supplementary material for: Serine synthesis and catabolism in starved lung cancer and primary bronchial epithelial cells
Source: Cancer Metab. 2024 Mar 21;12:9. doi: 10.1186/s40170-024-00337-3 (PMC10956291; doi:10.1186/s40170-024-00337-3)
Supplement: Supplementary file 1 — Additional file 1. [file 40170_2024_337_MOESM1_ESM.docx]

**Haitzmann et al, Supplementary Materials and Methods**

**Quantitative PCR (qPCR).** Total RNA was extracted with the Qiagen RNeasy Mini kit (Qiagen, Hilden, Germany) and reverse transcribed with the qScript™ cDNA Synthesis Kit (Quantabio, Beverly, MA, USA) according to the manufacturer’s instructions. The QuantiFast SYBR PCR kit (Qiagen) was used to perform qPCR on a LightCycler 480 (Roche, Vienna, Austria). Primers used were *PHGDH*, 5’*-*TGCAAATCTGCGGAAAGTGC-3’ (forward), 5’*-*GATGACATCAGCGGTCACCT-3’ (reverse); *PSAT1,* 5’*-*AAAAACAATGGAGGTGCCGC-3’ (forward), 5’*-*GGCTCCACTGGACAAACGTA-3’ (reverse), *SHMT1*, 5’*-*AGGAAAGGAGTGAAAAGTGTGGAT-3’ (forward), 5’*-*GACACCAGTGTCGCTCTGGATCTG-3’ (reverse); *SHMT2*, 5’*-*AGTCTATGCCCTATAAGCTCAACCC-3’ (forward), 5’*-* GCCGGAAAAGTCGAGCAGT-3’ (reverse); *MTHFD1*, 5’*-*TCTACACGAAGCAGGGCTTT-3’ (forward), 5’*-*GTCCAGGCATTGTGCTCATC-3’ (reverse); *MTHFD2*, 5’*-* GATGGCCTCCTTGTTCAGTTG-3’ (forward), 5’*-*ATCCTTGTCTGGAGAAACAGCATT-3’ (reverse); *ACTB,* 5’*-*ATTGCCGACAGGATGCAGGAA-3’ (forward), 5’*-*GCTGATCCACATCTGCTGGAA-3’ (reverse).

**Western blot.** Proteins were separated by sodium dodecyl sulfate-polyacrylamide gel electrophoresis using the Mini-PROTEAN® electrophoresis unit (BioRad, Hercules, CA, USA) and transferred to a PVDF membrane (BioRad). The following antibodies were used at the indicated dilutions overnight: PSAT1 (Abnova, Taipei City, Taiwan; H00029968-A01, 1:1000), PHGDH (Sigma-Aldrich, HPA021241, 1:1000). Blocking was performed in 5% bovine serum albumin (PHGDH) or 5% milk (PSAT1). Beta-actin was used as a loading control.

**Stable isotopic tracing.** Cells were washed two times with PBS and pre-treated with the respective starvation media for 24 hours to achieve a metabolic steady state. Thereafter, glucose, glutamine, serine or glycine were replaced by the indicated concentrations of ^13^C_6_-glucose (Cambridge Isotope Laboratories, Tewksbury, MA, USA), ^13^C_3_-serine (Cambridge Isotope Laboratories),^13^C_2_-glycine (Cambridge Isotope Laboratories) or 2 mM ^13^C_5_-glutamine (Sigma-Aldrich or Cambridge Isotope Laboratories) for additional 24 hours. After washing with saline, metabolism was quenched by immediately freezing the cells on liquid nitrogen.

**Sample extraction and gas chromatography-mass spectrometry (GC-MS) measurements.** Metabolite extraction, derivatization and analysis were performed as described (1). Metabolites were extracted on ice with cold 62.5% methanol in water, containing norvaline as an internal standard. . For analysis of conditioned media, 2 µl per sample were extracted. Cells were scraped, chloroform was added and the samples were sonicated. To extract metabolites, samples were centrifuged for 10 minutes at 4°C, and the phases were separated. For measurements of polar metabolites, the methanol in water phase was dried by vacuum centrifugation. For GC-MS, samples were derivatized with 20 mg/mL methoxyamine in pyridine (Thermo Fisher Scientific, Waltham, MA, USA) for 60 minutes at 37°C and thereafter with N-(tert-butyldimethylsilyl)-N-methyl-trifluoroacetamide (TBDMS, Thermo Fisher Scientific) for 30 minutes at 60°C.

Separation was performed with an Agilent 7890B GC system coupled to an Agilent 5977A Inert MS system (Agilent, Santa Clara, CA, USA). A DB35MS column flushed with helium as a carrier gas at a flow rate of 1 mL/minute was used. A volume of 1 µL sample was injected in splitless mode with an inlet temperature of 270°C. The GC oven was kept at 100°C for 3 minutes and ramped to 300°C with a gradient of 3.5°C/minute. Mass spectrometry was performed in Electron ionization (EI) mode at 70 eV by scanning the mass range m/z 100-605. El-Maven software (2) was used for peak quantification. Isotopologue distributions were corrected for natural abundance with IsoCor (3). Standards were run regularly to ensure proper metabolite identification. Absolute quantification was not performed. Metabolite amounts (peak areas) were normalized to internal standard and total protein.

**Gene expression analysis using publicly available datasets.** Gene expression data of SSP genes were retrieved from the publicly available TCGA lung adenocarcinoma (LUAD) and lung squamous cell carcinoma (LUSC) datasets using the UCSC Xena platform (https://xenabrowser.net[/](https://xenabrowser.net/)). TCGA gene expression data were generated by the TCGA Research Network ([https://](https://www.cancer.gov/tcga)www.cancer.gov/[tcga](https://www.cancer.gov/tcga)).

**References**

(1) Lorendeau, D., Rinaldi, G., Boon, R., Spincemaille, P., Metzger, K., Jager, C., et al. (2017) Dual loss of succinate dehydrogenase (SDH) and complex I activity is necessary to recapitulate the metabolic phenotype of SDH mutant tumors. Metab. Eng. 43, 187-197

(2) Agrawal S, Kumar S, Sehgal R, George S, Gupta R, Poddar S, et al. El-MAVEN: A Fast, Robust, and User-Friendly Mass Spectrometry Data Processing Engine for Metabolomics. Methods Mol Biol. 2019;1978:301-21

(3) Millard, P., Delepine, B., Guionnet, M., Heuillet, M., Bellvert, F., and Letisse, F. (2019) IsoCor: isotope correction for high-resolution MS labeling experiments. Bioinformatics 35, 4484-4487
