# Supplementary material for: Serine synthesis and catabolism in starved lung cancer and primary bronchial epithelial cells
Source: Cancer Metab. 2024 Mar 21;12:9. doi: 10.1186/s40170-024-00337-3 (PMC10956291; doi:10.1186/s40170-024-00337-3)
Supplement: Supplementary file 2 — Additional file 2: Supplementary Figure S1. Overall survival in lung adenocarcinoma (n=491) and squamous cell carcinoma patients (n=471) from the TCGA dataset expressing low or high levels of SSP or one-carbon metabolism enzymes. Median values were used as a cut-off. Survival analysis was performed using Log-rank test. Supplementary Figure S2. Cell counts and viability under starvation conditions. A Viable cell numbers and B the percentage of viable (calcein positive) cells after treatment with the respective starvation media for 36 hours. Results are mean +/- SEM from four independent experiments. Group comparisons were performed by Two-way ANOVA with Tukey post-hoc analysis. B As a positive cell death control, cells treated with serum-free medium lacking glucose, glutamine, serine and glycine was included. Viability was compared to the respective 10 mM glucose, high serine/glycine conditions using One-way ANOVA with Dunnett post-hoc analysis.*P < 0.05; **P < 0.01; ***P < 0.001; n.s., not significant; # versus high glucose; Ser/Gly, serine/glycine. Supplementary Figure S3. SSP gene expression in lung cancer and normal lung epithelial cells under starvation treatments. A mRNA levels of SSP and one-carbon metabolism genes in two PHGDH-positive cell lines, A549 and H1299 and normal bronchial epithelial cells (BEC) treated in medium containing high glucose and serine/glycine levels for 48 hours. Results are shown as mean +/- SEM from four (A549, H1299) or three (BEC) independent experiments; Group comparisons were performed by One-way ANOVA with Dunnett post-hoc analysis versus BEC for each gene.***P < 0.001. B-D Representative PHGDH and PSAT1 immunoblots and quantifications of blots from four (A549) or three (H1299 cells or BEC cells) experiments cultured in medium containing high (H) or low (L) serine/glycine, and high (H) or low (L) glucose for 48 2 hours (with medium replacement after 24 hours), with or without variations in cystine (high or low, only A549). β-acti [file 40170_2024_337_MOESM2_ESM.pdf]

Fig. S1

Adenocarcinoma

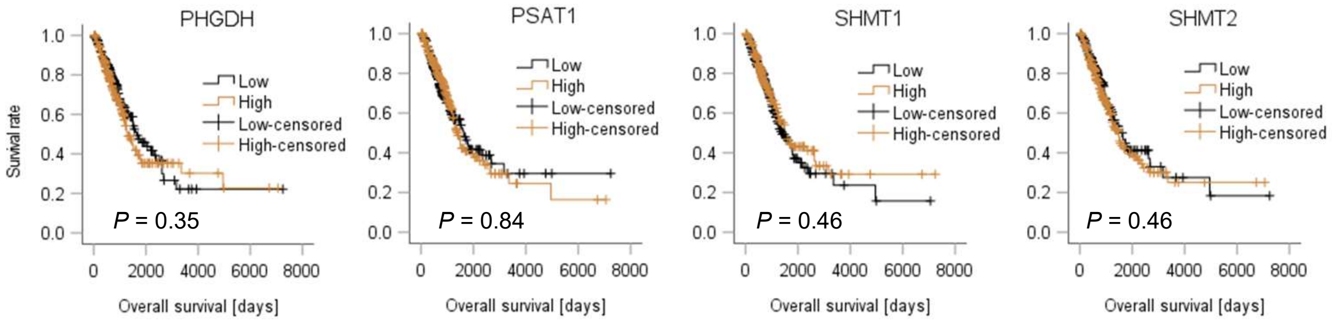

Squamous cell carcinoma

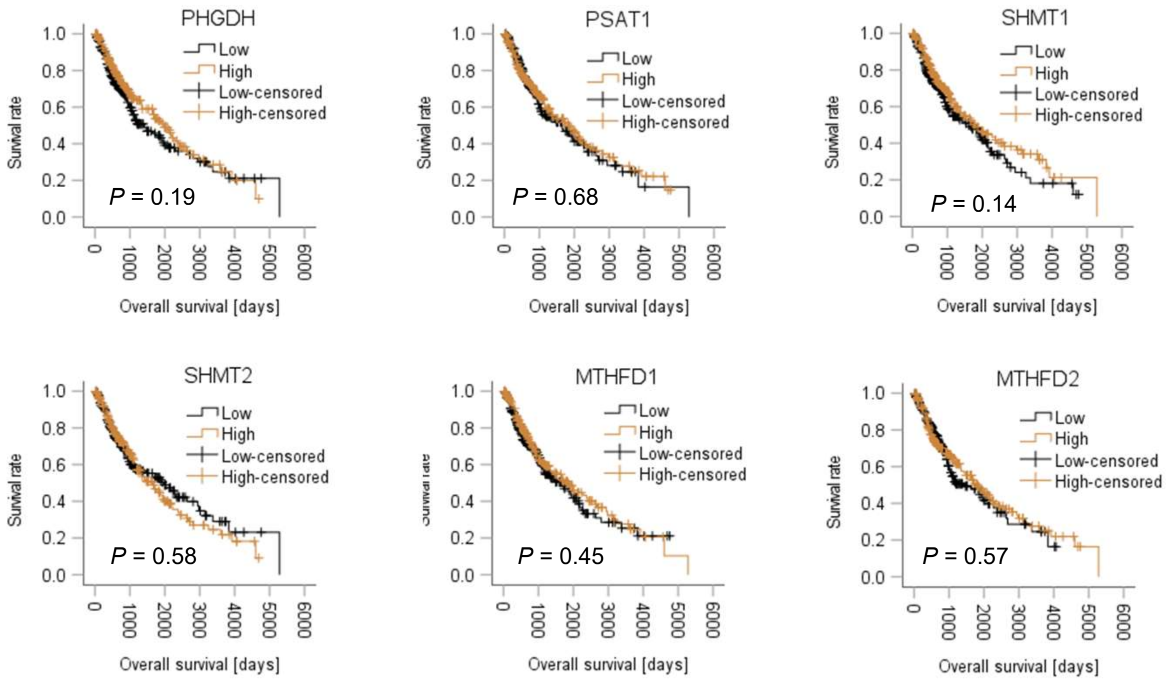

Fig. S2

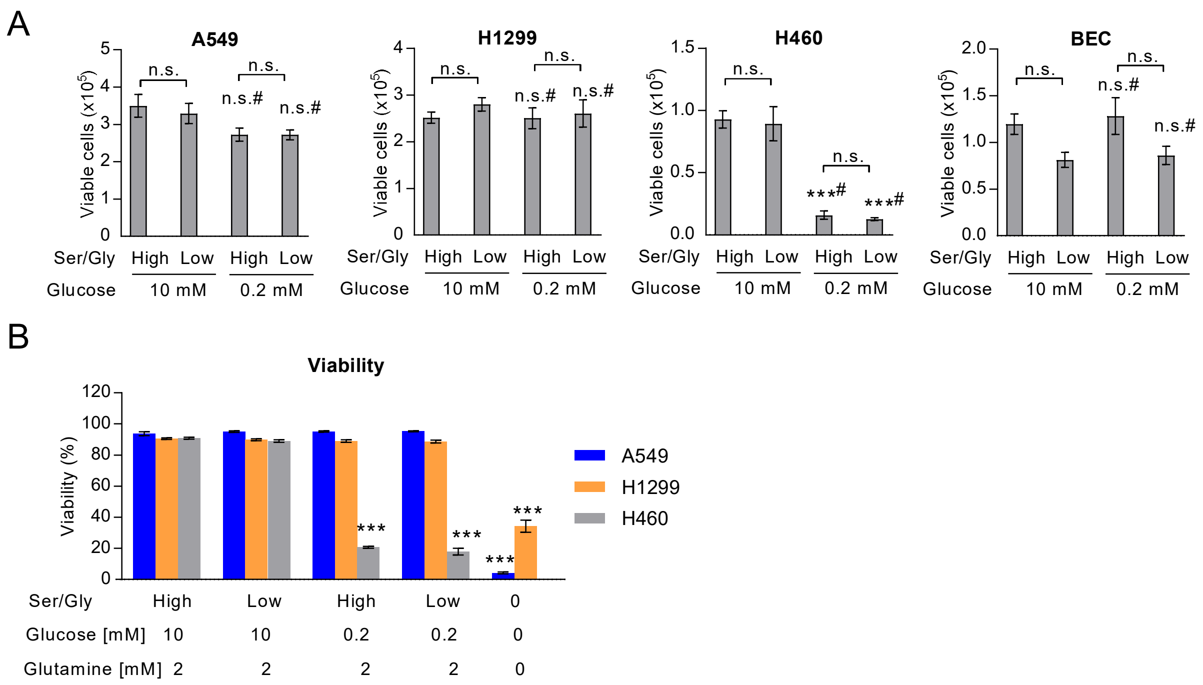
A
A549
H1299
H460
BEC
Viable cells (x10^5)
n.s.
n.s.#
***#
Ser/Gly
High
Low
Glucose
10 mM
0.2 mM
B
Viability
Viability (%)
***
A549
H1299
H460
Ser/Gly
Glucose [mM]
Glutamine [mM]

Fig. S3

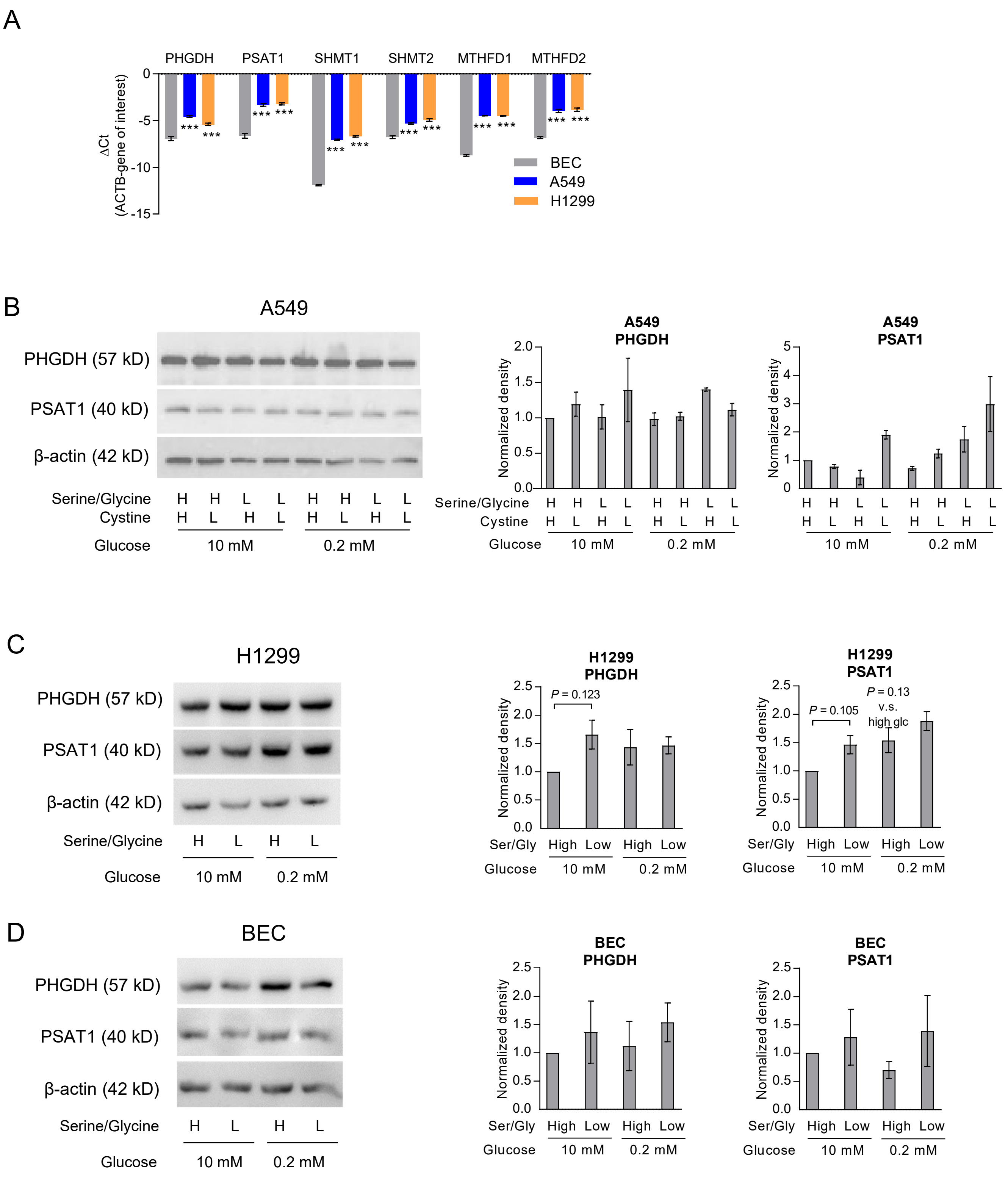
A
PHGDH
PSAT1
SHMT1
SHMT2
MTHFD1
MTHFD2
ΔCt
(ACTB-gene of interest)
0
-5
-10
-15
***
BEC
A549
H1299
B
A549
PHGDH (57 kD)
PSAT1 (40 kD)
β-actin (42 kD)
Serine/Glycine H H L L H H L L
Cystine H L H L H L H L
Glucose 10 mM 0.2 mM
A549
PHGDH
Normalized density
A549
PSAT1
C
H1299
Serine/Glycine H L H L
H1299
PHGDH
P = 0.123
H1299
PSAT1
P = 0.105
P = 0.13
v.s.
high glc
Ser/Gly High Low High Low
Glucose 10 mM 0.2 mM
D
BEC
BEC
PHGDH
BEC
PSAT1

Fig. S4

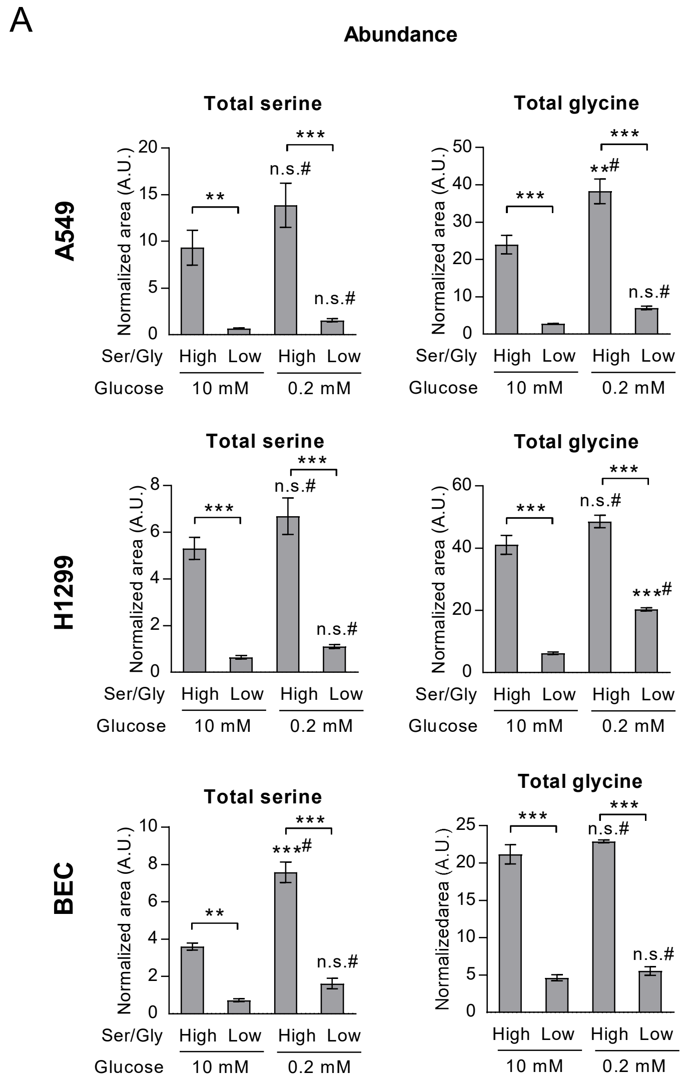
A
Abundance
A549
Total serine
Total glycine
H1299
Total serine
Total glycine
BEC
Total serine
Total glycine
Normalized area (A.U.)
Normalizedarea (A.U.)
Ser/Gly
High Low High Low
Glucose
10 mM
0.2 mM
**
***
n.s.#
**#
***#

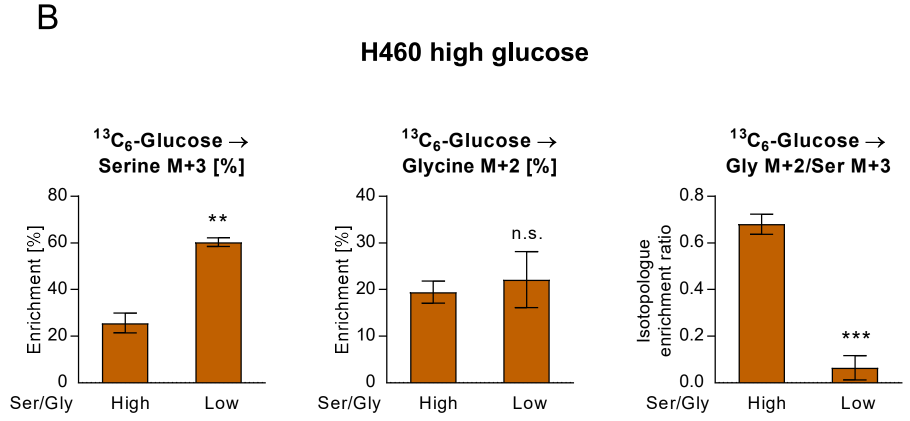
B
H460 high glucose
$^{13}C_6$-Glucose → Serine M+3 [%]
$^{13}C_6$-Glucose → Glycine M+2 [%]
$^{13}C_6$-Glucose → Gly M+2/Ser M+3
Enrichment [%]
Isotopologue enrichment ratio
Ser/Gly
High
Low
**
n.s.
***

Fig. S5

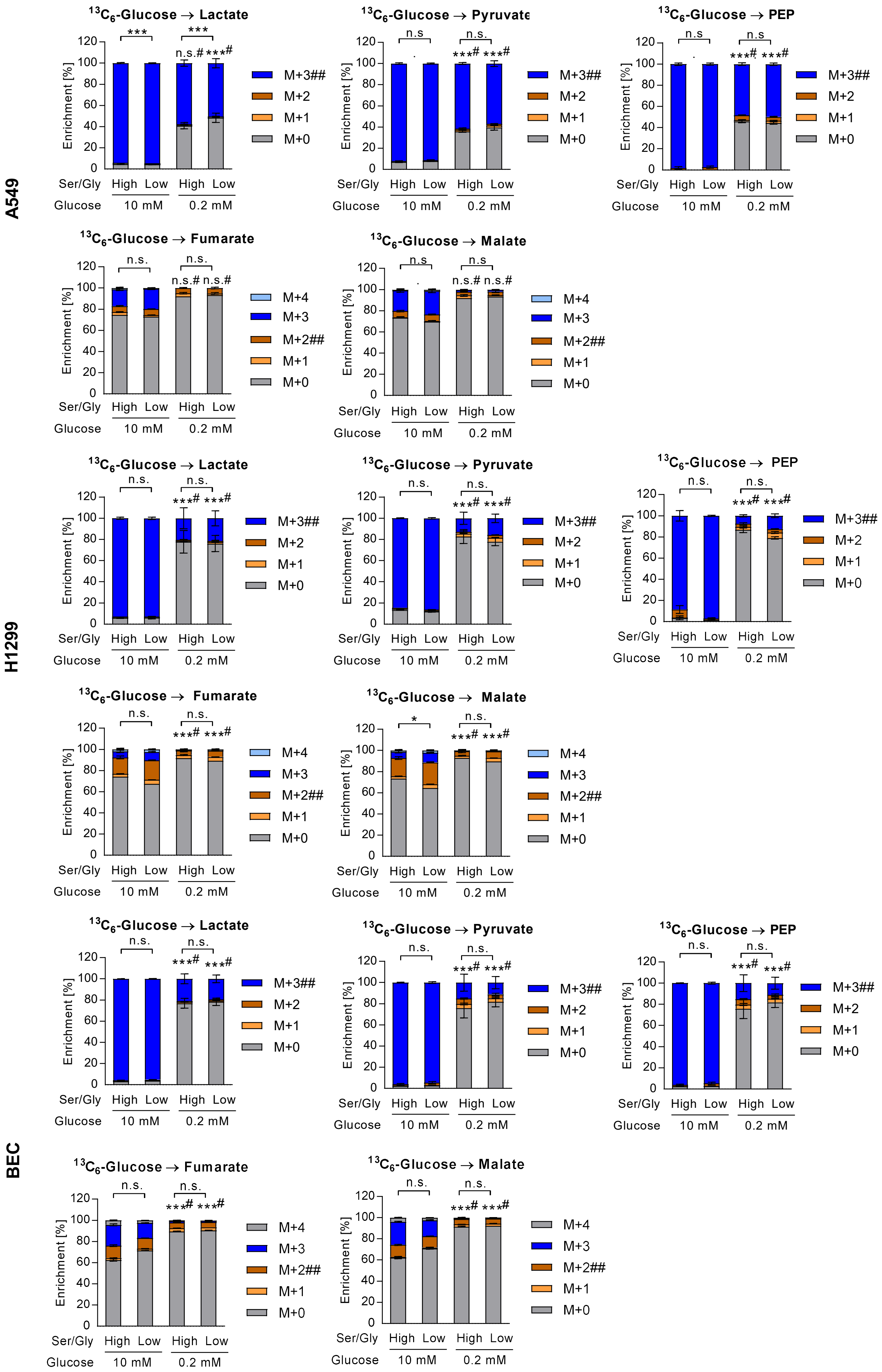
A549
H1299
BEC
$^{13}C_6$-Glucose → Lactate
$^{13}C_6$-Glucose → Pyruvate
$^{13}C_6$-Glucose → PEP
$^{13}C_6$-Glucose → Fumarate
$^{13}C_6$-Glucose → Malate
Enrichment [%]
Ser/Gly High Low High Low
Glucose 10 mM 0.2 mM
M+4
M+3##
M+3
M+2##
M+2
M+1
M+0
n.s.
***
*
n.s.#
***#

Fig. S6

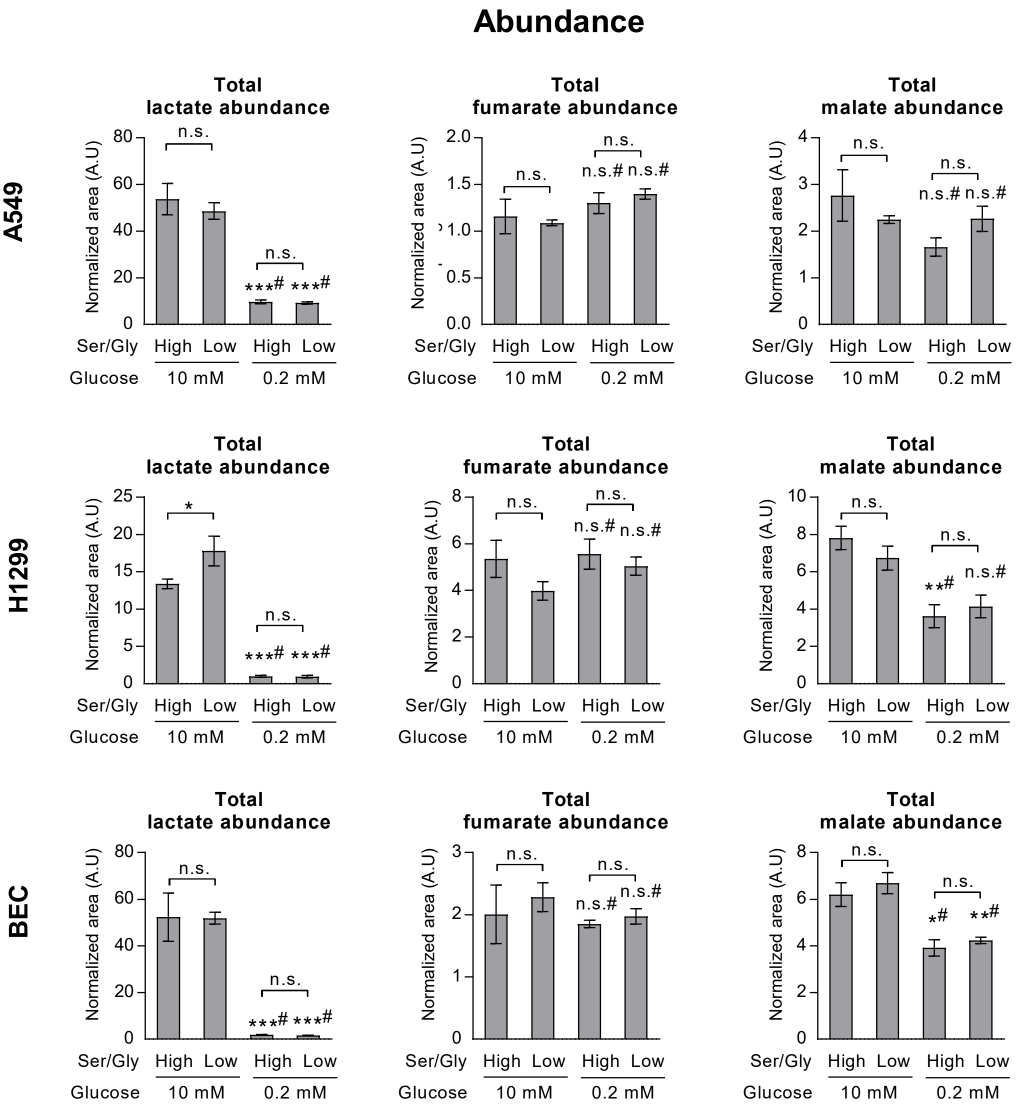
Abundance
A549
H1299
BEC
Total lactate abundance
Total fumarate abundance
Total malate abundance
Normalized area (A.U)
Ser/Gly
High
Low
Glucose
10 mM
0.2 mM
n.s.
n.s.#
*
**#
***#
*#

Fig. S7

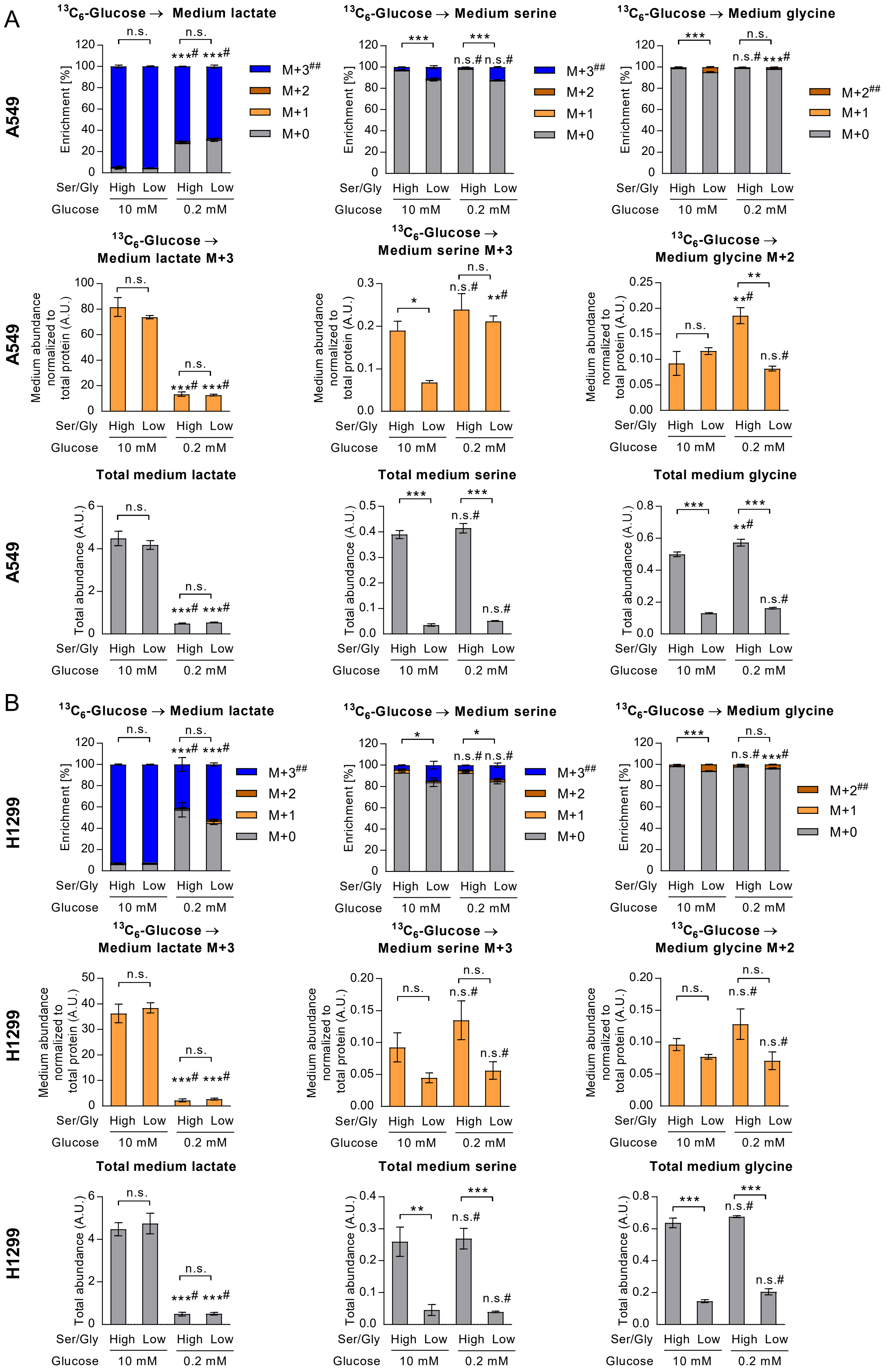

A
B
A549
H1299
13C6-Glucose → Medium lactate
13C6-Glucose → Medium serine
13C6-Glucose → Medium glycine
13C6-Glucose → Medium lactate M+3
13C6-Glucose → Medium serine M+3
13C6-Glucose → Medium glycine M+2
Total medium lactate
Total medium serine
Total medium glycine
Enrichment [%]
Medium abundance normalized to total protein (A.U.)
Total abundance (A.U.)
M+3##
M+2
M+2##
M+1
M+0
Ser/Gly High Low High Low
Glucose 10 mM 0.2 mM

Fig. S8

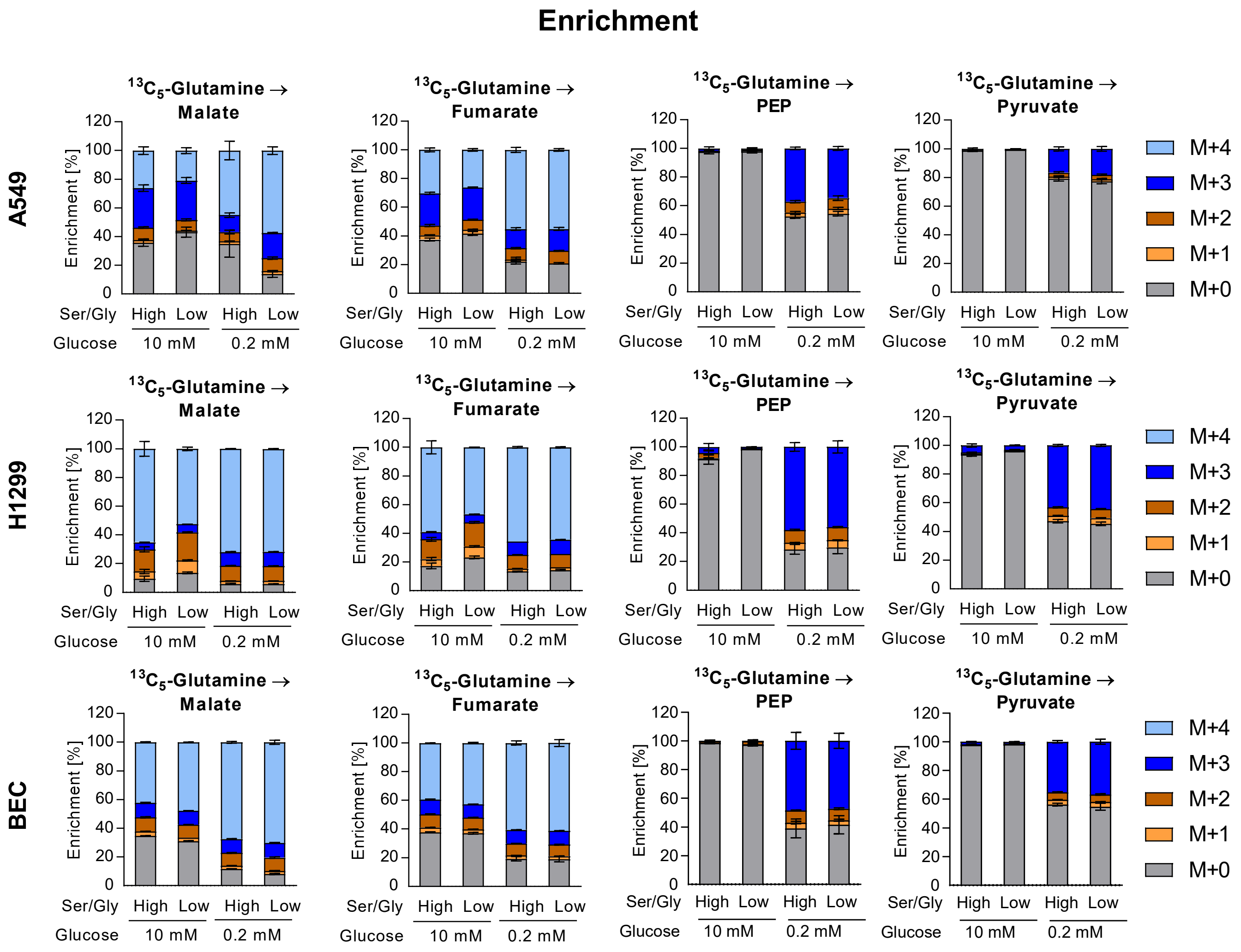
Enrichment
A549
H1299
BEC
$^{13}C_5$-Glutamine → Malate
$^{13}C_5$-Glutamine → Fumarate
$^{13}C_5$-Glutamine → PEP
$^{13}C_5$-Glutamine → Pyruvate
Enrichment [%]
0
20
40
60
80
100
120
Ser/Gly
High
Low
High
Low
Glucose
10 mM
0.2 mM
M+4
M+3
M+2
M+1
M+0

Fig. S9

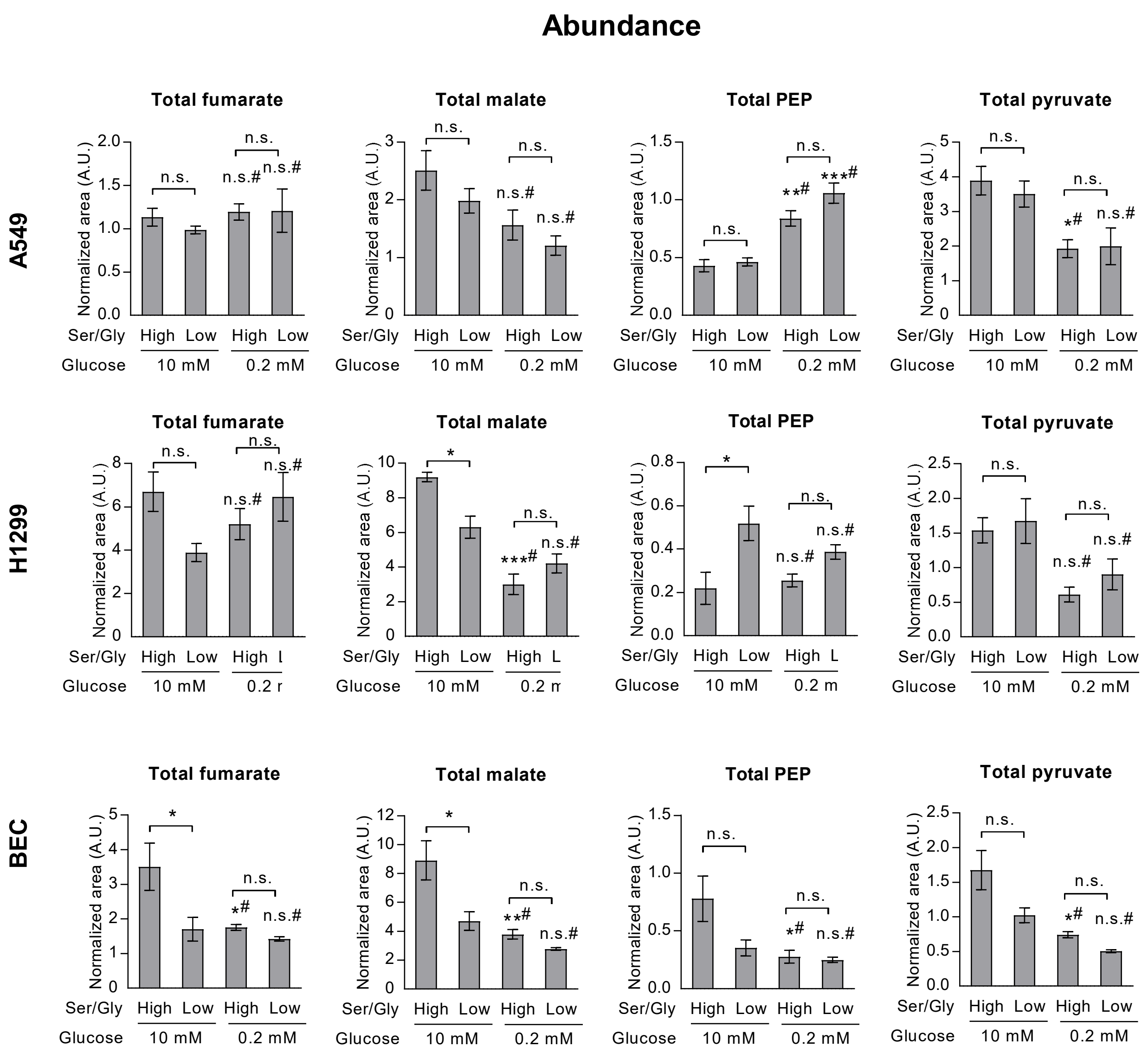
Abundance
A549
H1299
BEC
Total fumarate
Total malate
Total PEP
Total pyruvate
Normalized area (A.U.)
Ser/Gly
High
Low
Glucose
10 mM
0.2 mM
n.s.
n.s.#
*
**#
***#
*#

Fig. S10

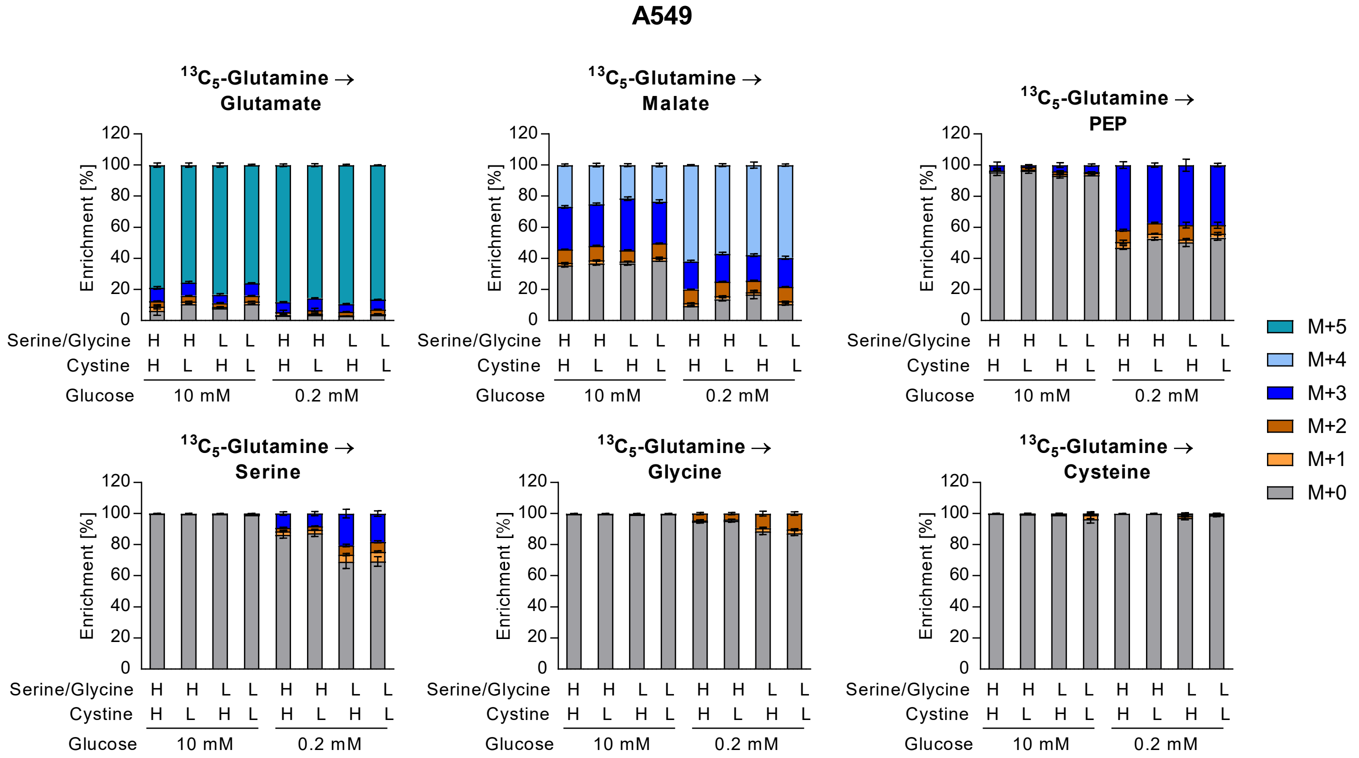
A549
$^{13}C_5$-Glutamine → Glutamate
$^{13}C_5$-Glutamine → Malate
$^{13}C_5$-Glutamine → PEP
$^{13}C_5$-Glutamine → Serine
$^{13}C_5$-Glutamine → Glycine
$^{13}C_5$-Glutamine → Cysteine
Enrichment [%]
0
20
40
60
80
100
120
Serine/Glycine H H L L H H L L
Cystine H L H L H L H L
Glucose 10 mM 0.2 mM
M+5
M+4
M+3
M+2
M+1
M+0
