# Supplementary material for: Serine synthesis and catabolism in starved lung cancer and primary bronchial epithelial cells
Source: Cancer Metab. 2024 Mar 21;12:9. doi: 10.1186/s40170-024-00337-3 (PMC10956291; doi:10.1186/s40170-024-00337-3)
Supplement: Supplementary file 3 — Additional file 3. [file 40170_2024_337_MOESM3_ESM.docx]

Table S1. Correlations of serine synthesis and one-carbon metabolism genes with ASNS in lung adenocarcinoma (LUAD) and lung squamous cell carcinoma (LUSC).

|  | LUAD | | LUSC | |
| --- | --- | --- | --- | --- |
| Gene expression | Correlation coefficient^1^ | *P* | Correlation coefficient^1^ | *P* |
| PHGDH / ASNS | **0.48** | < 0.001 | **0.43** | < 0.001 |
| PSAT/ ASNS | **0.69** | < 0.001 | **0.71** | < 0.001 |
| PSPH/ ASNS | **0.53** | < 0.001 | **0.45** | < 0.001 |
| SHMT1 / ASNS | **-0.094** | 0.032 | **0.21** | < 0.001 |
| SHMT2 / ASNS | **0.53** | < 0.001 | **0.50** | < 0.001 |
| MTHFD1 / ASNS | **0.30** | < 0.001 | 0.08 | 0.08 |
| MTHFD2 / ASNS | **0.61** | < 0.001 | **0.71** | < 0.001 |

Significant correlations are highlighted in bold. ^1^Spearman's rank correlation coefficient
