# Supplementary material for: Serine synthesis and catabolism in starved lung cancer and primary bronchial epithelial cells
Source: Cancer Metab. 2024 Mar 21;12:9. doi: 10.1186/s40170-024-00337-3 (PMC10956291; doi:10.1186/s40170-024-00337-3)
Supplement: Supplementary file 4 — Additional file 4. [file 40170_2024_337_MOESM4_ESM.pdf]

Supplementary material: uncropped Western blots

A

Fig. 1A

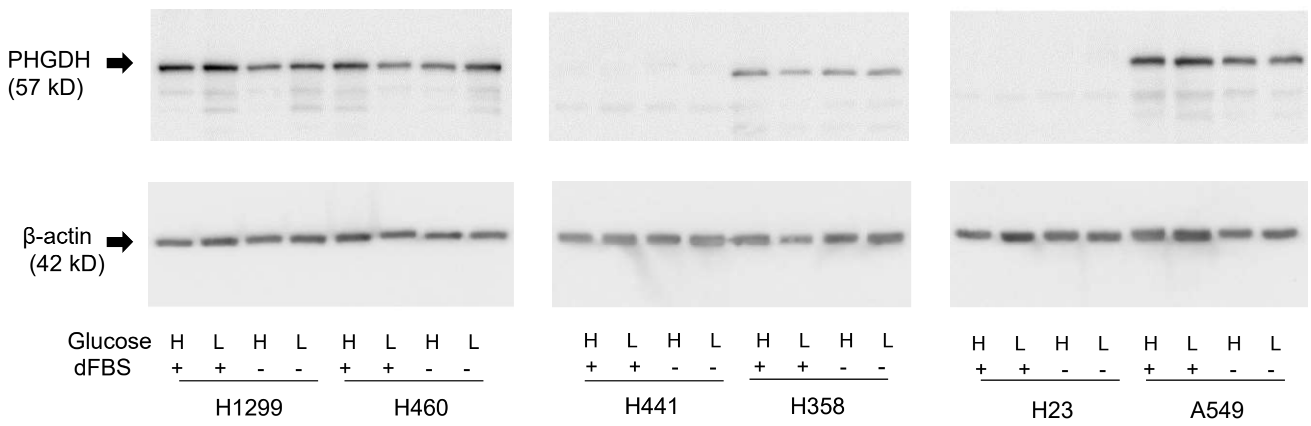

B

Fig. S3B

A549

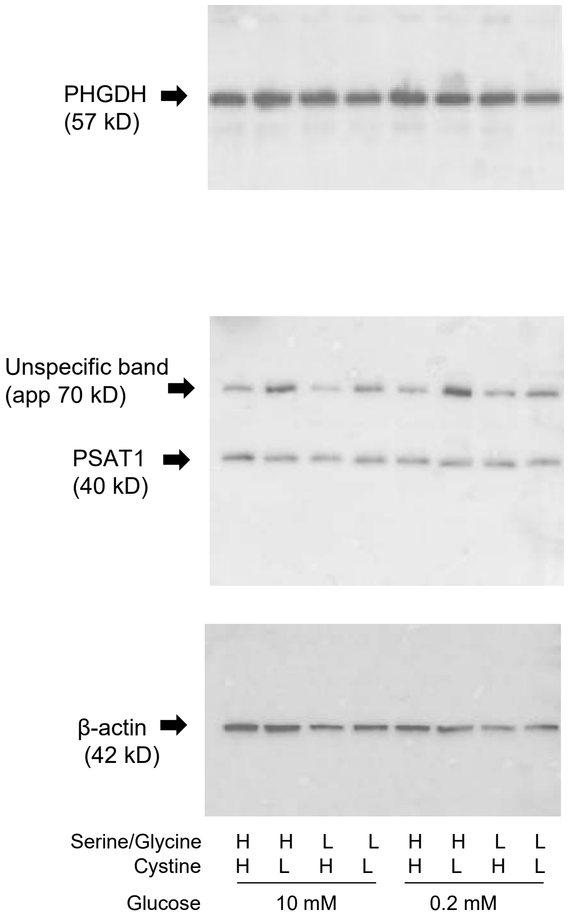

C

Fig. S3C

H1299

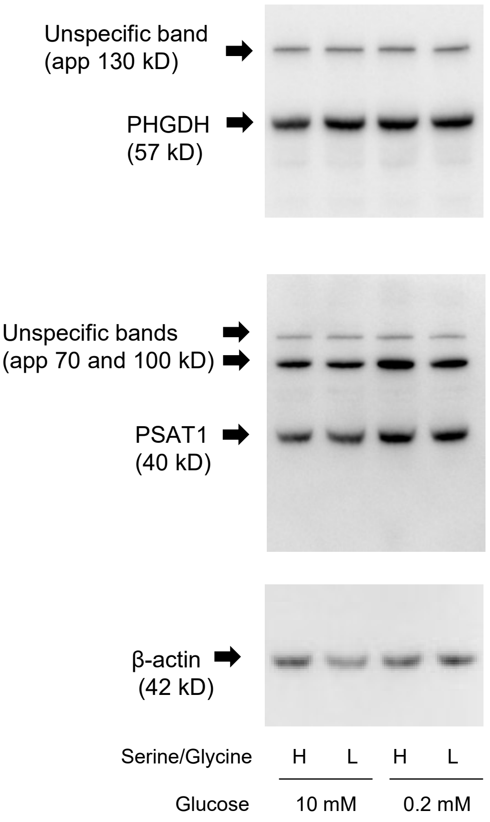

Supplementary material: uncropped Western blots (continued)

D

Fig. S3D

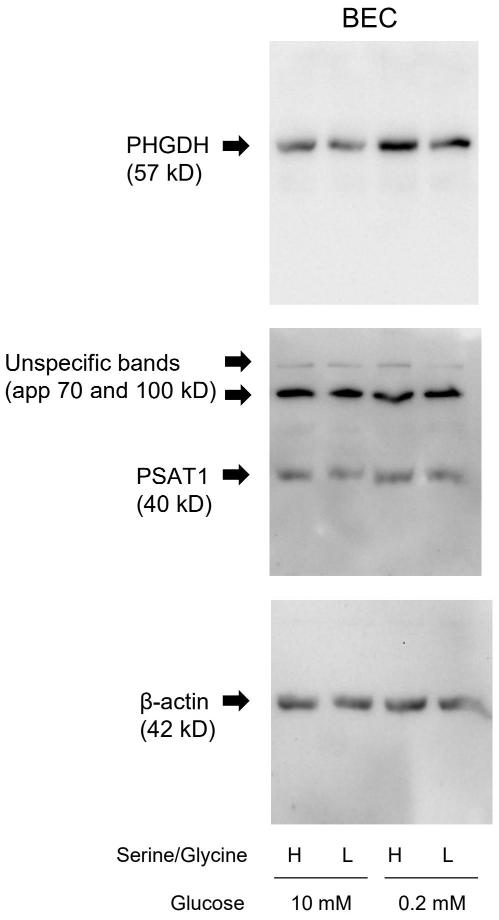

Uncropped immunoblots. A Immunoblots from Fig. 1A. PHGDH expression in lung cancer cell lines treated with 10 mM (high, H) or 0.2 mM (low, L) glucose with or without dialyzed serum (dFBS). Beta-actin was used as a loading control. **B-D** Immunoblots from Supplementary Figure S3. A549, H1299 cells or BEC were cultured in medium containing high (H) or low (L) levels of serine/glycine, and high (H) or low (L) levels of glucose for 48 hours, A549 were additionally treated with high or low concentrations of cystine (Cys). β-actin was used as a loading control.
